# Global mapping of transcription factor motifs in human aging

**DOI:** 10.1371/journal.pone.0190457

**Published:** 2018-01-02

**Authors:** David Alfego, Ulrich Rodeck, Andres Kriete

**Affiliations:** 1 School of Biomedical Engineering, Science and Health Systems, Drexel University, Philadelphia, Pennsylvania, United States of America; 2 Department of Dermatology and Cutaneous Biology, Thomas Jefferson University, Philadelphia, Pennsylvania, United States of America; Universitat des Saarlandes, GERMANY

## Abstract

Biological aging is a complex process dependent on the interplay of cell autonomous and tissue contextual changes which occur in response to cumulative molecular stress and manifest through adaptive transcriptional reprogramming. Here we describe a transcription factor (TF) meta-analysis of gene expression datasets accrued from 18 tissue sites collected at different biological ages and from 7 different in-vitro aging models. In-vitro aging platforms included replicative senescence and an energy restriction model in quiescence (ERiQ), in which ATP was transiently reduced. TF motifs in promoter regions of trimmed sets of target genes were scanned using JASPAR and TRANSFAC. TF signatures established a global mapping of agglomerating motifs with distinct clusters when ranked hierarchically. Remarkably, the ERiQ profile was shared with the majority of in-vivo aged tissues. Fitting motifs in a minimalistic protein-protein network allowed to probe for connectivity to distinct stress sensors. The DNA damage sensors ATM and ATR linked to the subnetwork associated with senescence. By contrast, the energy sensors PTEN and AMPK connected to the nodes in the ERiQ subnetwork. These data suggest that metabolic dysfunction may be linked to transcriptional patterns characteristic of many aged tissues and distinct from cumulative DNA damage associated with senescence.

## Introduction

The analysis of transcriptomes has become an important tool to study aging-associated processes, but has yet to deliver consistent datasets across tissues and experimental platforms. Gene expression studies comparing tissues from flies, worms, mice and humans have revealed tissue- and organism-specific aging profiles [1], with commonalities in gene ontology classifications centered around metabolism, specifically mitochondrial function [2, 3]. A recent comprehensive assessment of gene expression profiles in tissues has confirmed the diversity of gene expression profiles in human aging [4]. To what extent cellular heterogeneity, epigenetics or stochastic processes play a role in this diversity is unknown [5–7]. Another unresolved issue is the relevance of replicative in-vitro senescence to biologically aged tissues [8–10]. Specifically, in-vitro replicative senescence represents a permanent post-mitotic state with a specific gene expression pattern whereas fibroblasts isolated from very old donors (>90 years) retain mitotic potential [11, 12]. In one study, no senescence-associated transcripts were found in human tissues [13]. Furthermore, it is unclear to which extent other experimental platforms reveal molecular alterations relevant to biologically aged tissues, for example cells from patients suffering from Progeria syndromes, rare genetic disorders characterized by symptoms of premature aging [14, 15].

To gain a better understanding of changes in the transcriptome associated with aging in these different settings, we performed a transcription factor (TF) meta-analysis across multiple tissue datasets derived from tissues aged in-vivo as compared to experimental in-vitro aging models. Prior TF analyses of the aging process have been limited to specific TFs including Forkhead box TFs (FOXOs), signal transducer and activators of transcription (STATs), E2 family TFs (E2F) or nuclear factor kappa-b (NF-κB) [16–20]. These TFs participate in a wide range of cellular functions, yet present only a small fraction of all potentially relevant TF proteins.

Alternatively, TF activities can be estimated from gene expression data [21–23]. To interrogate age-associated changes in TF activities across experimental platforms we scanned promoter regions of differentially expressed target genes using TF position weight matrices (PWM) or motifs, provided by JASPAR and TRANSAC [24, 25]. The task required comparative analysis of gene expression datasets from diverse tissues and experimental studies, both in reference to study design and platforms. A number of techniques have been developed to harmonize otherwise incompatible gene expression data, such as re-annotations, re-scaling, median rank scoring and supervised classifications across datasets [26, 27]. However, limited overlap of transcripts between cells, tissues or studies in aging restricts transcript harmonization [2, 5]. Secondly, inclusion of smaller experimental studies with less statistical rigor hinder application of a uniform significance thresholds required by meta-analyses [3, 28]. Methods of abstracting from specific transcripts and expression values include (i) gene set enrichment, (ii) gene ontologies and (iii) transcription factor analyses, exploring shared commonalities in gene function, ontology or regulation, respectively. Thus, transcription factor analyses provide a method to decipher commonalities in transcriptional regulation based on prioritized target genes independent of specific platforms. Shorter lists may reduce potential false positives, specifically in experimental studies [29, 30], but enrichment scores will be more significant if more transcripts are taken into account. Here, a minimum number of transcripts was estimated with respect to the strength of rank correlation analyses, which are an essential method deployed in this study to determine similarities between samples.

Since transcription factors can both activate and repress genes based on cell type, cellular context and a complex dynamic of TF interactions [31], we performed a sign-less approach targeting both, the most up- and down-regulated target genes. Finally, to distinguish aging phenotypes, we restricted our subsequent comparative analyses of TF signatures on ranks to further diminish variances with respect to experimental design differences. This analysis was carried out using consistently trimmed gene expression data from 18 datasets portraying biological aging in different human tissues and 7 experimental cell aging studies and. Our analysis revealed three distinct tissue groups, which can be aligned with TF signatures of specific experimental models: classical replicative senescence in proliferative cells, senescence compared to quiescence excluding the influence of cell cycle, and an energy restriction model in quiescence (ERiQ), i.e. forced restriction of ATP supply. Furthermore, we identified subsets of unique motifs distinct for senescence and energy restriction. These anti-correlating motifs appeared to be enriched in one phenotype and avoided in the other. Our results suggest the existence of distinct gene regulatory phenotypes contributing to the aging process in response to DNA damage or metabolic stress in-vivo and in-vitro.

## Results

### Agglomerative hierarchical clustering

**Fig 1:**
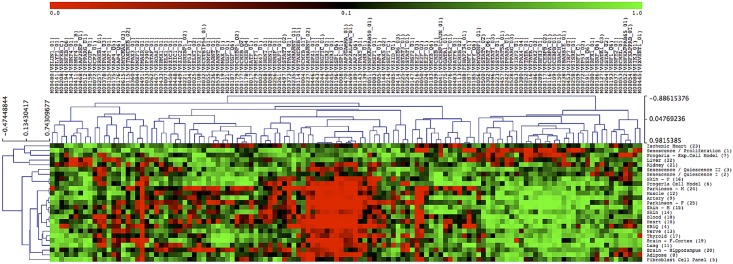
Global mapping of transcriptional regulation in human aging. Enrichment scores of prioritized transcription factor motifs, using TRANSFAC database, are visualized as a dendrogram. The heat map represents unique gene regulatory signatures of 18 human tissues and 7 cell aging experiments. Motifs enriched in aging (p<0.05) are indicated in red, and avoided motifs in green. Motifs are ranked and sorted by hierarchical clustering using Spearman rank correlation, complete linkage. Tissue samples agglomerate with either an experimental energy restriction cell model in quiescence (ERiQ, sample #4), or anti-correlate as a group of nine senescence related samples. The corresponding analysis using JASPAR motifs is provided in S1 Fig.

We generated transcription factor signatures representing 18 tissues collected at different biological ages and 7 experimental aging models. Datasets comparing young and aged human tissues included adipose, artery, heart, lung, muscle, nerve, skin, thyroid and blood [4], brain [32], kidney [33] and liver [34]. Additional tissue samples were from a second set of male and female skin samples [35], age-matched ischemic heart samples as positive control for senescence [36] and age-matched male and female Parkinson’s brain tissues as positive controls for an energy restricted phenotype [37]. Cell lines and experimental models included senescence in comparison to proliferating and quiescent fibroblasts [38], Progeria cells [15], an experimental Progeria model [39], a panel of fibroblast cell lines from donors of different age [40] and an energy restriction model in quiescence (ERiQ) [41, 42]. Depending on the study design (comparing two conditions or cross-sectional), differentially expressed genes had been ranked by regression analysis or fold-change, and these lists were trimmed to the top 75 up- and 75 downregulated transcripts. Details about the data location, age-ranges, statistical methods, and p-values obtained after further trimming published lists, are provided in S1 File. The resulting 150 genes were then analyzed for transcription factor enrichment, using JASPAR and TRANSFAC catalogues. The resulting global maps consisted of 125 TRANSFAC and 376 JASPAR enriched motifs and provided an overview of transcriptional regulation in our samples (Fig 1, S1 Fig). Maps were organized as dendrograms to reveal differences in subsets of transcriptional motifs across samples. Complete linkage clustering emphasizes dissimilar members and there was a considerable anti-correlation of both motifs and samples independent of the catalog used. Samples agglomerated either with the experimental energy restriction model (ERiQ) or with in-vitro senescence, but showed gradual differences in the senescence group.

### Classification of gene regulatory signatures

**Fig 2:**
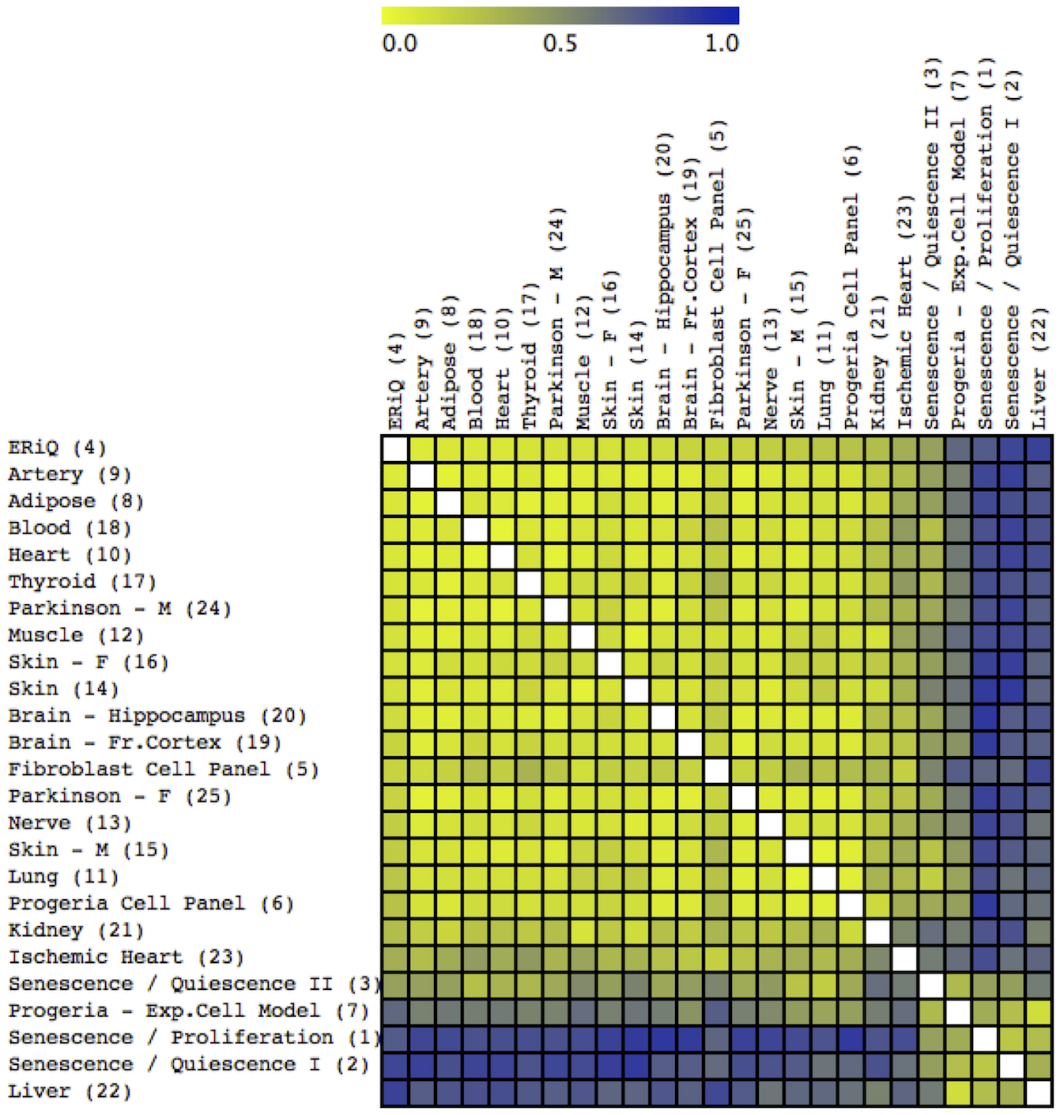
Sample distance map and classification. The distance map indicates similarities of samples based on ranked transcription factor enrichment scores, using 125 TRANSFAC motifs. Dissimilarity increases from yellow to blue. The distance map is overlaid by a result from K-Means Classification (KMC), discriminating three distinct groups marked by white lines. Most samples included in this study aggregate with an experimental energy restriction model and also include brain samples from Parkinson’s patients. In contrast, tissues including kidney, liver, female skin and ischemic heart aggregate with experimental models of senescence.

**Fig 3:**
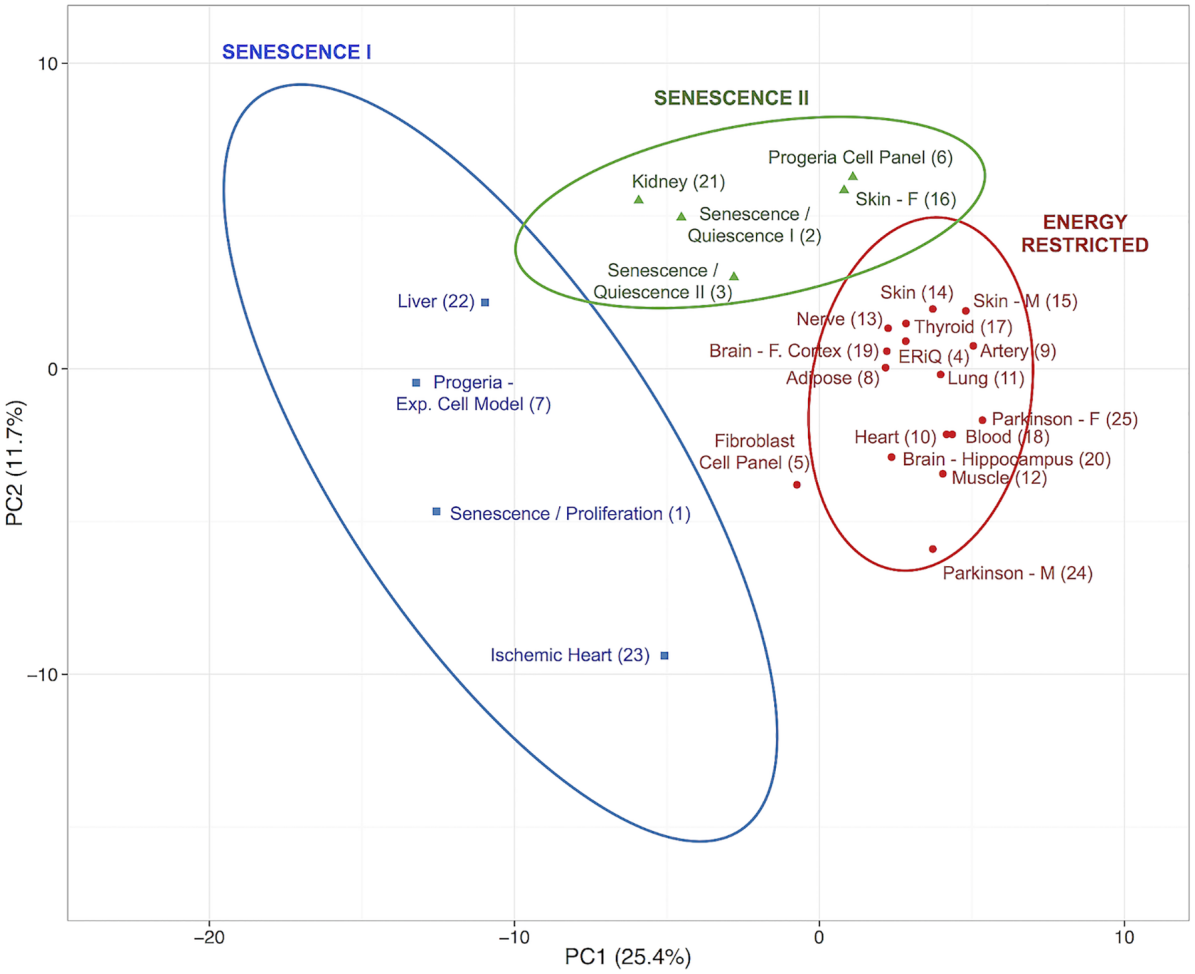
Principal component analysis of aging samples using transcription factor signatures. The first two components of the principle component analysis (PCA) using enrichment scores of transcription factor motifs are shown. The first two components account for the largest variance of 25.4% and 11.7% across sample signatures. Three distinct groupings are depicted, consistent with the classification shown in Fig 2. Group boundaries represent 80% likelihood for samples to be found in each specific group.

A set of classification methods was carried out to group samples according to ranked motifs. Self-organizing maps (SOM) and K-means Nearest Neighbor clustering (KMC) revealed three distinct phenotypical groups, shown here in combination with a sample distance matrix (Fig 2). A Principal Component Analysis (PCA) of ranked motifs confirmed these three distinct groups of signatures (Fig 3). The first group included most aged tissues including adipose tissue, artery, brain (frontal cortex and hippocampus), heart, lung, muscle, skin and brain tissues from Parkinson’s patients. The experimental ERiQ model is closely aligned with this group, whereas the fibroblast panel of cells from donors of different ages extends outwards to senescence. All other samples clustered with senescent in-vitro cell models as common denominators. The first of these (Figs 2 and 3) clustered the in-vitro replicative senescence model together with liver, ischemic heart tissue and the Progeria cell model. A second senescence cluster contained two datasets comparing in-vitro senescence to quiescent cells, the Progeria cell panel, kidney and female skin tissue.

### Anti-correlating transcription factors

We noticed specific motifs contributing to an anti-correlating pattern in the complete-linkage dendrograms (Fig 1), emphasizing differences between senescence and energy restriction phenotypes. We selected a senescence data set comparing senescence with quiescence (#2), to exclude the influence of cell cycle and to provide a focus on DNA damage, and compared this set with the ERiQ data (#4). Short lists of motifs were derived after ranking enrichment scores, and the combined list of 48 TRANSFAC and 133 JASPAR motifs was further filtered for switching behavior. Motifs showing enrichment (p < 0.05) in both conditions were removed. Of the remaining 14 motifs, 8 TFs were enriched in ERiQ, but avoided in senescence, and 6 TFs were enriched in senescence (Table 1), but avoided in ERiQ. The contribution of these motifs to our sample classifications was indicated by a non-parametric significance test of microarrays (SAM), using three sample groups as shown in Fig 3, and/or high loads in the PCA, with an absolute load value >0.05 in at least one of the first two principle components.

**Table 1:** Switching transcription factor motifs.

*Motifs*	*ERiQ / Quiescence*	*Senescence / Quiescence*	*Fold*	SAM(*)/ PCA(+)
***SWITCHING***				
M00516 (V$E2F_03) MA0469.1 (E2F3)	0.0002 (11)	0.5325 (86)	2.20E+3	*/+
MA0506.1 (NRF1)	0.0003 (30)	0.6401 (198)	2.07E+3	*
MA0006.1 (Ahr::Arnt) M00237 (V$AHRARNT_02)	0.0002 (23)	0.3247 (125)	2.02E+3	*/+
M00245 (V$EGR3_01) MA0732.1 (EGR3)	0.0002 (10)	0.2649 (49)	1.46E+3	*/+
M00185 (V$NFY_Q6) M00287 (V$NFY_01) M00288 (F$HAP234_01)	0.0011 (20)	0.9841 (93)	9.23E+2	*/+
M00246 (V$EGR2_01)	0.0005 (17)	0.2694 (50)	5.54E+2	*/+
M00005 (V$AP4_01)	0.0204 (34)	0.8207 (115)	4.02E+1	+
MA0018.1 (CREB1) M00114 (V$TAXCREB_01)	0.0498 (85)	0.8563 (258)	1.71E+1	*/+
***SWITCHING OPPOSITE***				
MA0106.2 (TP53)	0.9909 (279)	0.0002 (1)	4.26E+3	*
MA0914.1 (ISL2)	0.9058 (225)	0.0117 (7)	7.76E+1	+
MA0474.1 (Erg)	0.9082 (229)	0.0192 (15)	4.73E+1	*
M00280 (V$RFX1_01)	0.6231 (90)	0.0189 (4)	3.29E+1	+
MA0479.1 (FOXH1)	0.9602 (252)	0.0321 (23)	2.99E+1	*/+
MA0861.1 (TP73)	0.8834 (216)	0.0479 (30)	1.84E+1	+

Scores of enriched transcription factor motifs and their ranks (in parentheses) are provided which switch between energy starvation and senescence (compared to quiescent cells). The upper section of rows lists those TFs enriched in ERiQ (p<0.05) but avoided in senescence (p>0.25). The lower section represents the opposite. Variant TF motifs exhibiting similar trends in scores are listed. Ratios of differences in enrichment scores and indicators of additional significance in SAM and/or PCA loads are included.

### Protein-protein interactions

**Fig 4:**
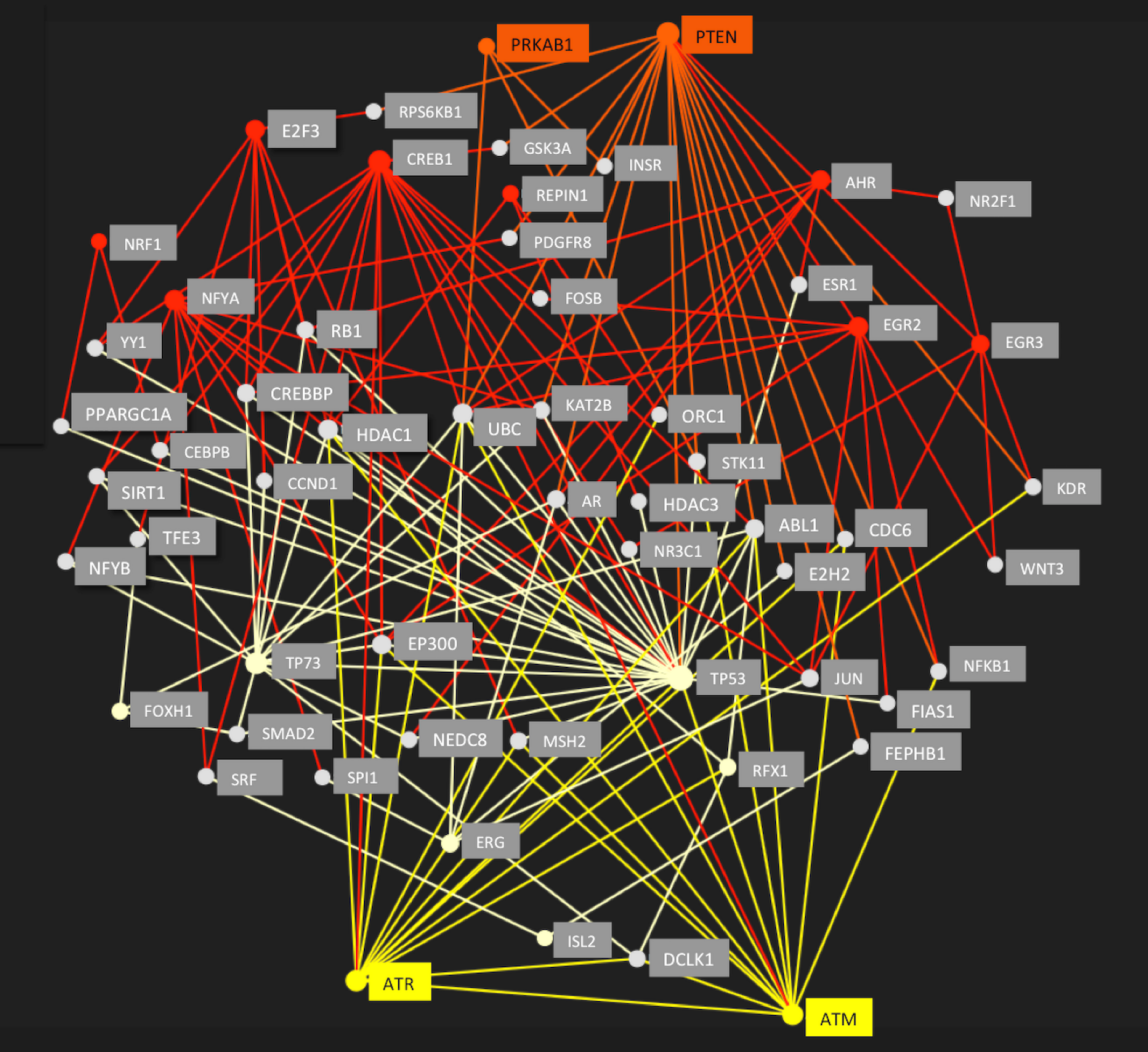
Regulatory protein-protein-interaction (PPI) network. The PPI network was seeded by 14 enriched transcription factor proteins (see Table 1), which switch between enrichment in senescence (light yellow nodes and edges) and enrichment in energy restriction (red nodes and edges). Connectivity was predicted by STRING, and visualized with NetworkAnalyst. All nodes were probed for their connectivity to DNA stress sensors ATM (ataxia-telangiectasia mutated) and ATR (ATM- and Rad3-Related) in yellow, and multiple connections were found to the subnetwork associated with senescence, but not to the energy restriction nodes. In contrast, nodes enriched in energy restriction revealed strong connectivity to the energy sensors PRKAB1 (AMPK) and phosphatase and tensin homolog (PTEN) in orange, while these proteins did not connect to the senescence nodes. The combined network shown here suggests involvement of intermediate proteins (gray nodes), some of which may also change activity as indicated by different colored network edges converging onto these nodes such as histone deacetylases HDAC1, HDAC3, SIRT1 and EP300 and polyubiquitin-C precursor (UBC). The network provides a flexible functionality allowing cells to adapt to different stressors.

The analysis of anti-correlated transcription factors seeded a minimalistic protein-protein interaction (PPI) network (Fig 4), with a total of 58 nodes and 150 edges. We tested the hypothesis that node-switching is caused by cellular responses to specific intracellular stressors. For the senescence dataset, we included ataxia-telangiectasia mutated (ATM) and Rad3-Related (ATR) protein hubs as proximal sensors for DNA damage response (DDR) [43]. For energy stress we had chosen phosphatase and tensin homolog (PTEN) and AMP-activated kinase (AMPK) as established proximal sensors [44, 45].

We observed an association of the ATM and ATR hubs with the senescence group of nodes. In contrast, PTEN and AMPK hubs connected to nodes associated with the energy restriction subnetwork, suggesting the existence of two specific subnetworks. The DDR pattern is connected to p53 and retinoblastoma protein (p53-Rb) pathway activation in senescence [46, 47]. Specifically, p53 is an enriched hub in the senescence subnetwork and RB1 is a predicted participating protein. Deacetylating Forkhead box (FOXO) transcription factors modulate transcripts involved in response to DNA damage [48] and FOXH1 was enriched in senescence. Phosphorylation of cAMP responsive element binding protein 1 (CREB1) by ATM correlates with a decrease in CREB transactivation potential and reduced interaction between CREB and its transcriptional coactivator, CREB-binding protein (CBP) [49]. Conversely, CREB1 is induced by mitochondrial dysfunction [50] and improves mitochondrial biogenesis along with nuclear respiratory factor 1 (NRF1) [51], and both of these transcriptional regulators were enriched in ERiQ.

The PPI network predicts a role of histone deacetylases (HDAC1, HDAC3, SIRT1) and histone acetyltransferase p300 (EP300 HAT) as additional modulators of transcriptional regulation in ERiQ. ATM can activate HDAC1, which suppresses E2F transcriptional activation, which are avoided motifs in senescence. HDACs are also recognized as important mediators of autophagy, requiring ubiquitination [52]. Activation of the ubiquitin-proteasomal system has been linked to DNA damage responses [53], and the PPI model provides a direct link of both ATR and ATM, as well as AMPK and PTEN to the polyubiquitin-C precursor UBC, suggesting the involvement of ubiquitination in both. NF-κB TF motifs such as M00054 (V$NFKAPPAB_01) from TRANSFAC, and MA0105.1 (NFKB1) and MA0778.1 (NFKB2) from JASPAR, were enriched in both senescence models and in ERiQ.

## Discussion

Aging may be viewed as a process requiring continuous adaptive responses to chronic cellular stress caused by cumulative molecular damage and energetic challenges. It is poorly understood how this process is reflected at the transcriptional level. The meta-analysis presented here provides pivotal insights into gene regulatory signatures in biologically aged tissues in comparison to experimental cell models of aging. To enable cross-comparison of heterogeneous datasets, we restricted our exploratory examination to trimmed sets of differentially expressed target genes derived from gene expression studies providing whole genome coverage. Inclusion of both up- and downregulated transcripts in combination with subsequent analyses of ranked motifs allowed inclusion of smaller experimental studies. Motif enrichments provide initial global maps of transcriptional regulation in human aging that are suitable to generate new hypotheses (Fig 1, S1 Fig). Despite a limited overlap of single genes between tissues, the most salient finding of this study is the prediction of three distinct cellular aging phenotypes, associated with either DNA damage-induced senescence or energy deprivation, whereby the former can be distinguished further by the influence of cell cycle motifs. For the promotor regions considered here, each of the tissue signatures can be associated with a specific experimental model (Table 2).

**Table 2:** Gene regulatory phenotypes.

Phenotype	Energy Restriction	Senescence	
**Experimental Cell Models**	Energy Restriction in Quiescence (ERiQ) (4) Fibroblast Cell Panel in Quiescence (5)	*Senescence w/o cell cycle (2*, *3)* Progeria Cell Panel (6)	Replicative Senescence (1) Progeria Exp. Model (7)
**Tissues**	Adipose (8) Artery (9) Brain (15, 19) Blood (19) Heart (10) Lung (11) Muscle (12) Nerve (13) Skin (14, 15) Thyroid (17) Parkinson (24, 25)	Kidney (21) Skin–Female (16)	Liver (22) Ischemic Heart (23)

The table summarizes three prevailing regulatory signatures identified in this study. Each tissue group is best represented by a specific experimental fibroblast model—energy restriction in quiescence, senescence compared to quiescence excluding the effects of cell cycle, and classical replicative senescence as it occurs in proliferating cell cultures. Numbers in parentheses provide sample IDs.

Senescence presents as a multi-stage, diversifying process rather than a static endpoint [54]. This diversity was evident in our classifications and allowed aggregation of senescent samples in two main groups. Comparing TF motifs in replicative senescence with proliferating cells showed enriched TF motifs not only involved in DNA damage, but also cell cycle regulation and cell fate, such as nuclear factor Y (NFY), paired-box (PAX), and activating transcription factor (ATF) [55–57]. The Progeria sample in this group was obtained from an experimental model comparing immortalized skin fibroblasts with cells carrying Progerin, a truncated version of Lamin A protein. Liver was one tissue related to this group (Figs 2 and 3). Telomere shortening, senescence and chronic inflammation are known hallmarks of liver aging [58, 59]. Furthermore, studies in mice have indicated increase of γ-H2AX foci in liver, a proxy marker of DNA damage, but not in post-mitotic heart and muscle [8]. This senescence cluster also included the ischemic heart, consistent with the accumulation of senescent fibroblasts after myocardial infarction [60].

The second senescence cluster aggregated samples lacking enrichment of cell cycle related motifs. Two experimental samples compared senescence with quiescent, growth factor-starved fibroblasts. Although both datasets were obtained with different platforms and analyses, they correlated closely in signatures and classifications (Figs 1, 2 and 3). The Progeria sample associated with this group compared proliferating control fibroblasts with proliferating HGPS fibroblasts, precluding analysis of the effect of quiescence on gene expression patterns. Tissues included in this senescent cluster were female skin and kidney, which have been associated with inflammation, senescence and differences in gender [18, 61, 62]. An experimental study on kidney aging had identified NF-κB and STAT TFs as transcriptional regulators [18], which were enriched in our JASPAR and TRANSFAC signatures.

There is an apparent lack of suitable experimental models for aging in post-mitotic tissues. Here we show that a major cluster of samples was associated with the energy-restricted (ERiQ) model described by us earlier [41, 42], which combined inhibition of glucose uptake with mitochondrial dysfunction in quiescent cells. ERiQ grouped together with adipose, artery, brain, blood, heart, lung, muscle, skin and Parkinson’s brain samples. The fibroblast panel included in this group, representing differences between quiescent cells from young and old male donors, clustered borderline. It has been suggested that this particular experimental platform may portray the in-vivo situation more closely, but comparable studies of fibroblasts aged in-situ had also found early markers of senescence [63, 64]. Within the energetically compromised cluster, there was a noticeable difference in signatures between the frontal cortex and hippocampal brain areas, as there was also a noticeable gender difference amongst this group. Similarly, female skin samples originating from a smaller study that compared young and old donors [35], were different to skin samples from males, which were in close proximity to the signature from a more comprehensive set of both male and female skin samples [4]. Both differences in hormone status and immune cell composition have been suggested to contribute to disparities in skin and brain aging [65, 66]. Gender differences were similarly apparent between samples from Parkinson’s brain, compared to age-matched controls. The etiology of Parkinson’s disease attributes this to mitochondrial dysfunction, bioenergetics failure and gender dimorphisms [67–69].

A sign-less transcription factor analysis as conducted here limits inferences to function. Sign-sensitive approaches, until now, can utilize only very small TF catalogs [21]. However, some conclusions on the inner functioning of the protein-protein network constructed by switching motifs using current experimental knowledge can be drawn (Fig 4). Switching motifs were either enriched in senescence, and avoided in energy starvation, or vice versa. One group of senescence-associated proteins, indicated by switching motifs, connected to ATM and ATR as proximal sensors for DNA damage response [43, 70], which network under DNA damage stress with the p53 pathway [71]. In contrast, a different subset of proteins was connected to energy stress sensors AMPK [44, 45] and PTEN [72, 73]. Suppressed PTEN stimulates the Akt pathway, consistent with experimental findings of increased Akt signaling in ERiQ [41]. Increased Akt protein activity, but reduced p53 expression, are specific hallmarks of the ERiQ phenotype. Furthermore, interaction of Akt with CREB and FOXO transcription factors support a pro-survival cell state under energy stress [74, 75]. The exact function of HDACs and HATs in senescence [76, 77] or in response to low ATP [78, 79], as predicted by the PPI, still needs to be determined. In summary, senescence and energy restriction motifs relate to specific chronic stress sensors in distinct regulatory networks, causing anti-correlating clusters when ranked.

The mechanisms of transcription are complex and fine-tuned by cell specific transcriptional networks, epigenetics, methylation and ubiquitination [80–82], cell turnover rates [83] and heterogeneities in the functional elements of transcription [84]. Moreover, mitochondrial dysfunction and retrograde response mechanisms have been identified as factors influential for changes in gene expression [85–87]. Therefore, it is reasonable to assume that aging interferes with the transcriptional machinery in multiple ways, increasing diversity and heterogeneities. This may contribute to a mosaic of cellular phenotypes in some tissues. For instance, energy stress may play a primary role in tissues maintaining proliferative capacity, such as skin, but mitochondrial dysfunction has also been considered as an entry point into replicative senescence [88, 89]. Nevertheless, we were able to identify distinct gene regulatory signatures that share prevailing gene regulatory patterns during aging and demonstrate the validity of different experimental fibroblast models addressing these phenotypes. Specifically, our findings emphasize the role of mitochondrial dysfunction and energetic stress in post-mitotic tissues [90–93], involving AMPK and PTEN previously associated with longevity [94, 95]. Our results support the utility of transcription factor analyses in aging and application to tissues, experimental models and cell types. The transcription factors and related proteins identified here provide additional experimental targets for future in-depth analyses of transcriptional regulation in aging.

## Material and methods

### Gene selection and TF analysis

To include experimental studies in our analysis, we consistently trimmed published lists from all 25 samples of our panel to the 150 most significant differentially expressed genes per sample (75 up- and 75 down-regulated), improving statistical significance, as provided in S2 File. Trimming lists generally reduces enrichment scores in transcription factor analyses, but ranks of motifs are less affected. Specifically rank correlations, used here as a preferred clustering method to group samples, remain highly correlated (r > 0.93) when the number of transcripts is trimmed down from 250 to 150, but correlations drop below 0.9 when less than 100 transcripts are included (S2 Fig). We used PSCAN, 2016 build, to scan promoter regions between -450 bp upstream to 50 bp downstream of the transcription start site in the direction of transcription [96]. The motivating choice of this search range is the presence of highly expressed transcript clusters as revealed by aggregation plots [84]. PSCAN considers the highest enrichment score matching a transcription factor motif in each gene of a set. The degree of over- or underrepresentation of motifs is assessed by a z-test, associating each motif with a probability p of obtaining the same score in a random set of transcripts taken from the entire genome [96]. Included were 282 TRANSFAC [25] and 636 JASPAR motifs [24] derived from SELEX, SELEX-HT and ChIP-Seq data [97], and no additional annotated target sites were included. TF motif scores with significant p-values (FDR < 0.05) were considered enriched, and motifs with large p-values avoided. Motifs that did not reach significance in any sample (approximately 30%) were removed. The remaining set was further trimmed by a variance filter (Var>0.08) to accentuate motifs of dissimilar enrichment profile across samples.

### Clustering and switching motifs

To further reduce potential influences of experimental designs, TF scores within each profile were ranked. The resulting signatures of motifs were clustered hierarchically using Spearman Rank Correlation with complete linkage aggregation, emphasizing dissimilarities. The resulting dendrograms provided a global map of transcription factor regulation in human aging. In addition, ranked motif signatures were classified by non-parametric K-means nearest neighbor clustering (KMC), self-organizing maps (SOM), sample-distance maps and principal component analysis (PCA) to identify motif loads and detect dominant phenotypical patterns of regulation between samples. Tools to execute these analyses were provided in TM4/MeV [98] and ClustVis [99].

Significant motifs characterizing groups, along with PCA loads, were further filtered to identify a subset of switching TFs between replicative senescence compared to quiescence and the energy restriction (ERiQ) data set. We required an opposing, greater than 15-fold change in scores between senescence and ERiQ, with a score of at least p < 0.05 in one, and a change in rank. Predictions for transcription factor protein-protein-interactions were performed with NetworkAnalyst [100], referencing STRING [101], and connections required a confidence score of 500 and experimental evidence. A minimalistic PPI network was constructed within NetworkAnalyst, seeded by 14 switching TF nodes, and the resulting network was probed subsequently for connectivity with sensors for DNA damage, Ataxia-Telangiectasia Mutated (ATM) and ATM- and Rad3-Related (ATR), and for energy stress, phosphatase and tensin homolog (PTEN) and AMP-activated kinase (AMPK/PRKAB1).

## Supporting information

S1 Fig Clustering of JASPAR motifs. (PDF)

S2 Fig Enrichment scores. (PDF)

S1 File Gene expression sources. (PDF)

S2 File Gene expression data. (PDF)
