# Supplementary material for: Global mapping of transcription factor motifs in human aging
Source: PLoS One. 2018 Jan 2;13(1):e0190457. doi: 10.1371/journal.pone.0190457 (PMC5749797; doi:10.1371/journal.pone.0190457)
Supplement: S1 Fig — (PDF) [file pone.0190457.s001.pdf]

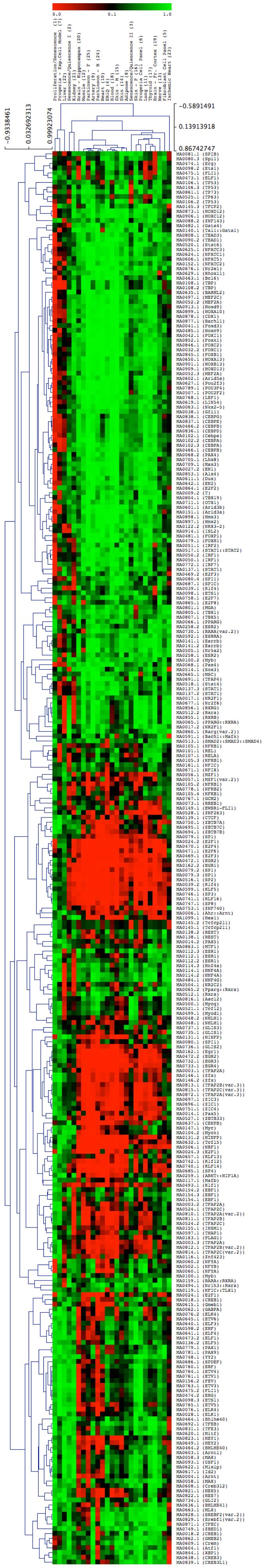

S1 Figure. Global map of transcription factor signatures in human aging using JASPAR

Hierarchical clustering of samples using signatures of transcription factor (TF) motif enrichment scores. Enrichment scores were determined for JASPAR motifs. Lower p-values in red represent enriched TFs ($p<0.05$) and higher in green represent avoided TFs. To determine similarities in signatures, a Spearman rank correlation was used. Following the trend in Fig. 1, correlation values range from +0.86 (positive correlation) to -0.59 (anticorrelation).
