# Supplementary material for: Global mapping of transcription factor motifs in human aging
Source: PLoS One. 2018 Jan 2;13(1):e0190457. doi: 10.1371/journal.pone.0190457 (PMC5749797; doi:10.1371/journal.pone.0190457)
Supplement: S2 Fig — (PDF) [file pone.0190457.s002.pdf]

S2 Figure

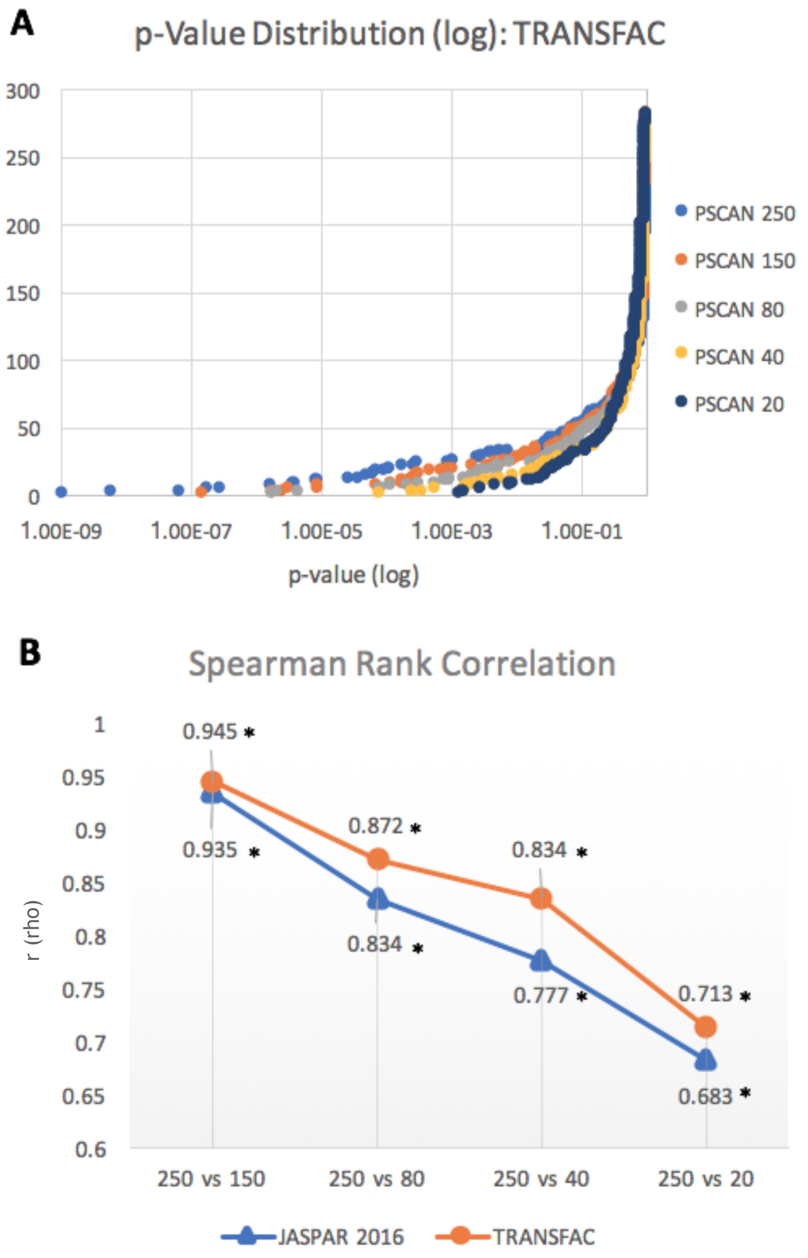

S2 Figure. Enrichment score distributions and Spearman Rank comparisons for differently sized gene sets.

A. Log distributions of the p-values representing transcription factor binding scores for a selection of 250, 150, 80, 40 or 20 genes, equally split for the most up- and down-regulated transcripts present in a transcriptome data set (using sample ERiQ, PSCAN and TRANSFAC motifs). As expected, the affinities followed an enrichment trend towards highly significant motifs in larger selections.

B. Spearman Rank Correlation comparing the largest selection (n=250) and each smaller one (SPSS package, Vers. 24, 2017). Shown is the correlation factor r (rho) obtained for comparisons of smaller sets against the largest set of 250 transcripts, using TRANSFAC and JASPAR motifs. High r values represents a good correlation between two sets and therefore minimal difference in rank correlations. This analysis confirmed our choice of 150 transcripts for all samples, with an r of .945 and 0.935 in TRANSFAC and JASPER, respectively ($p<0.0001$). While smaller sets would be desirable to reduce false positives, the correlation values drop significantly.
