# Supplementary material for: Global mapping of transcription factor motifs in human aging
Source: PLoS One. 2018 Jan 2;13(1):e0190457. doi: 10.1371/journal.pone.0190457 (PMC5749797; doi:10.1371/journal.pone.0190457)
Supplement: S1 File — (PDF) [file pone.0190457.s003.pdf]

S1 File. Gene Expression Sources

ID#	Cell/Tissue Type	Database ID	Age Range	Sample Size	Platform	Transcript Prioritization	Stat. Method	p-value Cutoff
1	Fibroblasts: Senescence/ Proliferation [1]	GSE60340	Immortalized Fibroblasts /Senescence	3/3 Cell Lines	Illumina HiSeq 2000	5x Fold	C	<1.05E-06
2	Fibroblasts: Senescence/ Quiescence I [1]	GSE60340	Immortalized Fibroblasts /Senescence	3/3 Cell Lines	Illumina HiSeq 2000	5x Fold	C	<1.05E-06
3	Fibroblasts: Senescence/ Quiescence II [2]	GSE15829	22-33/66 sen	5/1 Cell Lines	Codelink Human Whole Genome	4.5x Fold	C	<0.05
4	Fibroblasts: ERiQ [3]	GSE67981	22/Exp.	Exp. on Cell Line vs Control	Illumina HumanHT-12 V4.0	4x Fold	C	<0.05
5	Fibroblasts Cross-Section [2]	GSE15829	22-33/49-92	5/9 Cell Lines	Codelink Human Whole Genome	2.5x Fold	C	<0.05
6	Progeria I [4]	PMID15268757	8-16	7 Cell Lines	Affymetrix HG-U133A/B	4.1x Fold	D	NP
7	Progeria II [5]	GSE10123	hMSC	Exp. On Cell Line vs Control	Codelink Human Whole Genome	2.6x Fold	C	<0.05
8	Adipose (Subcut.) [6]	E-TABM-1140	20-70	94 Tissues	Combined Platforms, see Reference 6	Age Coeff.	A	<2.20E-05
9	Artery (Tibia) [6]	E-TABM-1140	20-70	112 Tissues	Combined Platforms, see Reference 6	Age Coeff.	A	<9.65E-08
10	Heart (L. Ventricle) [6]	E-TABM-1140	20-70	83 Tissues	Combined Platforms, see Reference 6	Age Coeff.	A	<3.46E-05
11	Lung [6}	E-TABM-1140	20-70	119 Tissues	Combined Platforms, see Reference 6	Age Coeff.	A	<2.85E-05
12	Muscle (Skeletal) [6]	E-TABM-1140	20-70	138 Tissues	Combined Platforms, see Reference 6	Age Coeff.	A	<6.55E-06
13	Nerve (Tibia) [6]	E-TABM-1140	20-70	88 Tissues	Combined Platforms, see Reference 6	Age Coeff.	A	<3.63E-05
14	Skin I (Lower Leg) [6]	E-TABM-1140	20-70	96 Tissues	Combined Platforms, see Reference 6	Age Coeff.	A	<5.28E-04
15	Skin II-a (M, Upper Arm) [7]	PMID23226273	25.8+/-5, 76+/-3.8	6/7 Tissues	Illumina BeadStation 500	1.5x Fold	C	<0.008
16	Skin II-b (F, Upper Arm) [7]	PMID23226273	26.7+/- 4, 70.7 +/-3.3	7/4 Tissues	Illumina BeadStation 500	1.5x Fold	C	<0.004
17	Thyroid [6]	E-TABM-1140	20-70	105 Tissues	Combined Platforms, see Reference 6	Age Coeff.	A	<1.04E-03
18	Blood [6]	E-TABM-1140	20-70	156 Tissues	Combined Platforms, see Reference 6	Age Coeff.	A	<1.76E-06
19	Brain (Frontal Cortex) [8]	PMID15190254	26-106	30 Tissues	Affymetrix HG-U95Av2	1.9x Fold	B	<0.005
20	Brain (Hippocampus) [9]	GSE46706	16-102	134 Tissues	Affymetrix Human Exon 1.0 ST	p-value	B	<0.01
21	Kidney [10]	PMID15562319	19-41/72-77	5/7 Tissues	Affymetrix HG-U133A/B	Age Coeff.	B	<3.72E-06
22	Liver [11]	GSE61260	19-41/72-77	5/7 Tissues	Affymetrix Human Gene 1.1 ST Array	3.5x Fold	C	<0.005
23	Ischemia: Heart, L. Ventricle [12]	GSE57345	> 18	Ischemia Heart/ NF 231	Illumina HiSeq 2000 / Affymetrix Human Exon ST1.1	1.3x Fold	C	<0.0025
24	Parkinson's (M)[13]	PMID20111594	68-89	6 cont. / 7 PD	Affymetrix HG-U133A/B	p-value	D	<0.05
25	Parkinson's (F) [13]	PMID20111594	68-89	3 cont. / 3 PD	Affymetrix HG-U133A/B	p-value	D	<0.05

Supplemental Table 1: Human Cell and Tissue Datasets. The table lists all human tissue and disease datasets used to evaluate gene expression through aging. References below describe sample collection and original data analysis per tissue. Not all data was available via Gene Expression Omnibus, but the Database ID references where all raw data may be obtained (data not in a deposit is referenced by PubMed PMID number). Age range represents the minimum and maximum ages of samples used to compare (NP – not provided) and the sample size describes the number of patients used in the original study. Specific platform was also noted per tissue study to be aware of any slight differences in the expression analysis. Our own analysis focused on the further prioritization of the published data by either a provided age coefficient, p-value (<0.05) or fold. Fold values provided here represent the least amount of fold change considered. For the studies included, age-related gene expression patterns had been identified using different statistical methods (A-D). For method A, a linear regression model between older and younger samples was performed with Benjamini Hochberg (BH) adjustment, whereas method B did not include such adjustment. Method C employed a two-sample comparison t-test between young and old samples and Method D used a Significance Analysis of Microarrays (SAM) as a method of analysis for fold and FDR according to platform. Folds/p-value cutoffs reflect the more stringent criteria applied here.

References and Analysis Methods

1. Purcell, M., A. Kruger, and M.A. Tainsky, *Gene expression profiling of replicative and induced senescence.* Cell Cycle, 2014. **13**(24): p. 3927-37.
   --RNA-seq was performed on immortal LFS MDAH041 cells that were young (PD 10-12), aged (PD 17-19) or replicatively senescent (PD28-30), as well as spontaneously immortal cells and cells that were induced into senescence or quiescence, in order to determine pathways common in all 4 types of senescence. Raw data analysis performed separately by us via Kallisto to compare senescence vs. control and quiescence vs. senescence. In GSE60340, cells can be identified as quiescent (samples Qui 1-3), proliferating/immortal (samples 1-3) and senescent (MDAH041 fibroblasts samples Nat 1-3).

2. Kriete, Andres, et al., *Cell autonomous expression of inflammatory genes in biologically aged fibroblasts associated with elevated NF-kappaB activity.* Immunity & Ageing, 2008 5(1): p. 5.
   --Human fibroblast cultures, established from skin samples derived from young and old donors, were obtained from the NIA Aging Cell Repository. After test and gene analysis, replicate readouts were averaged and normalized for differences between chips and outliers were detected. Expressions of characterized genes related to immunity and inflammation were identified and differential expressions determined. A variance filter trimmed the resulting list. The senescent sample was from a 66 year old donors and was compared to the group of young donors (22-33) in quiescence.

3. Yalamanchili, N., et al., *Distinct cell stress responses induced by ATP restriction in quiescent human fibroblasts*. Frontiers in genetics, 2016. 7.
   --A human fibroblast culture (AG10803, Coriell Institute for Medical Research, Camden, NJ) used in this study was derived from a 2 mm punch biopsy taken from the abdomen of a young donor. In order to introduce a energy starvation phenotype intracellular ATP levels were reduced, in quiescent cells, by simultaneously applying 2-Deoxy-d-glucose (2DG) and Carbonyl cyanide 4-(trifluoromethoxy)phenylhydrazone (FCCP) inhibitors. After gene analysis, data sets were normalized and outliers were excluded. Fold changes between four treated samples from two experiments relative to two samples from a quiescent control were determined, further filtered by a t-test between readouts and removal of unknown/predicted transcripts. From the resulting list of 2310 transcripts (fold changes >2, p < 0.005) only 75 of the highest induced and repressed transcripts (fold changes >3.4) were selected.

4. Csoka, A.B., et al., *Genome-scale expression profiling of Hutchinson-Gilford progeria syndrome reveals widespread transcriptional misregulation leading to mesodermal/mesenchymal defects and accelerated atherosclerosis.* Aging Cell, 2004. **3**(4): p. 235-43.
   --Cell lines used were obtained from Coriell Cell Repositories. For Affymetrix, all the chips were scaled to the same target intensity of 500. All possible pairwise correlation coefficients above 0.9 were computed. Significance Analysis of Microarrays (SAM) method was used to provide gene-specific t-tests and FDR.

5. Scaffidi, P. and T. Misteli, *Lamin A-dependent misregulation of adult stem cells associated with accelerated ageing.* Nat Cell Biol, 2008. **10**(4): p. 452-9.
--hTERT-TetOff-Pro cell lines expressing inducible GFP–wt-lamin A and GFP–progerin were generated by sequentially infecting hTERT-immortalized wild-type skin fibroblasts. To induce protein expression, the concentration of doxycycline was reduced. RNA labelling, hybridization on GE Healthcare CodeLink Gene Expression Bioarrays (Human Whole Genome) and data analysis were performed by GenUs Biosystems. RNA samples were collected at 0, 5 and 10 d after doxycycline removal. Two biological replicates for each time point were analyzed. Intensity values after hybridization were normalized to the median intensity of the chip and ratios between 5-d and 10-d time points to 0 d were calculated for each gene in either progerin- or wild-type lamin A-expressing cell lines. Genes showing at least two-fold differences in either cell line were selected for further analysis. For quantitative analysis of hMSC differentiation and quantitative RT-PCR, three biological replicates were analyzed and statistical significance of the differences was estimated using the one-tailed *t*-test.

6. Yang, J., et al., *Synchronized age-related gene expression changes across multiple tissues in human and the link to complex diseases.* Sci Rep, 2015. **5**: p. 15145.
--The GTEx pilot study collected a total of 9365 tissue samples targeting more than 30 distinct tissues from 237 post-mortem donors, combining experimental platforms for gene expression, RNA sequence and genotype analyses (see dbGaP Study Accession**:** phs000424.v3.p1 for listing of platforms). Yang et al. provided p-values from a linear regression model based on age (coefficient), gender and genotype. They performed the false discovery rate (FDR) adjustment on the p-values using Benjamini Hochberg method and an FDR less than 0.05 was used as the significance threshold throughout the paper unless otherwise specified.

7. Makrantonaki, E., et al., *Identification of biomarkers of human skin ageing in both genders. Wnt signalling - a label of skin ageing?* PLoS One, 2012. **7**(11): p. e50393.
--Full-thickness skin biopsies were resected from the sun-protected inner side of the upper arm of volunteers. Skin samples were provided by a total of 24 donors, all without any inflammatory or endocrinological disorders. The skin samples were obtained from European Caucasian young and elderly females and males. Chip hybridizations, washing, Cy3-streptavidin staining, and scanning were performed on an Illumina BeadStation 500 platform. All basic expression data analysis was carried out using the manufacturer's software BeadStudio 1.0 (Illumina). Raw data were background-subtracted and normalized using the 'rank invariant' algorithm, by which negative intensity values may arise. Normalized data were then filtered for significant expression on the basis of negative control beads.

8. Lu, T., et al., *Gene regulation and DNA damage in the ageing human brain.* Nature, 2004. **429**(6994): p. 883-91.
-- Dissections of the frontal pole were performed and tissue samples were snap frozen in liquid nitrogen. Total RNA was extracted and complementary RNA targets were prepared, labelled and hybridized with an Affymetrix Test 3 Array. Arrays were normalized and genes that correlated with age (Spearman rank correlation *P*-value <0.005) were determined and resolved by hierarchical clustering using dChip V1.3 software. Correlation coefficient analysis was performed to assess the relatedness of each case to every other case using S-PLUS 2000 software (Insightful Corp.). Gene-wise standardized expression values of the genes that show Spearman rank correlation with age were used to compute Pearson correlation coefficients between two cases. Significance analysis of microarrays (SAM) software was used to compare young (≤42 years old) and aged (≥73 years old) groups to determine the list of genes with a ≥1.5-fold change and median false discovery rate (FDR) <0.01.

9. Glass, Daniel, et al. "Gene expression changes with age in skin, adipose tissue, blood and brain." *Genome biology* 14.7 (2013): R75.
-- The hippocampal samples came from the UK Human Brain Expression Consortium used in this study were provided by the MRC Sudden Death Brain and Tissue Bank in Edinburgh and originated from 100 individuals (78 men and 22 women) of European descent. The tissues were profiled using the Affymetrix Human Exon 1.0 ST array (n=932 arrays) and then preprocessed using RMA using a high confidence list of probe sets. A linear mixed model was used to examine gene expression variability by age and confounding factors. A mixed-effects model was fitted in R. The *P*-values to assess significance for age effect were calculated from the Chi-square distribution with 1 degree of freedom using likelihood ratio as the test statistic. *P*-values were adjusted for multiple testing by controlling FDR with the Benjamini-Hochberg (B-H) procedure and using a threshold of 0.01. Enrichment analysis was carried out using the DAVID Bioinformatics Resource server with a significant level threshold of 0.05 in B-H corrected *P*-values.

10. Rodwell, G.E., et al., *A transcriptional profile of aging in the human kidney.* PLoS Biol, 2004. **2**(12): p. e427.
--Normal kidney samples were obtained either from biopsies of donor kidneys for transplantation or from nephrectomy patients (with informed consent). Key factors for each patient include sex, race, age, blood pressure, pathology, medications, serum creatinine, and urinary protein concentrations. Using the dChip program, microarray data were normalized according to the stable invariant set, and gene expression values were calculated using a perfect match model. The *p*-values used are based on *t*-tests and linear regression model.

11. Horvath, S., et al., *Obesity accelerates epigenetic aging of human liver.* Proc Natl Acad Sci U S A, 2014. **111**(43): p. 15538-43.
--Human liver samples from morbidly obese patients and healthy controls were analyzed by array-based mRNA expression profiling. Liver messenger RNA expression datasets from German patients were generated on the HuGene 1.1 ST gene array. The purpose of the study was to correlate these gene expression data with body mass index and with an epigenetic measure of age acceleration based on DNA methylation data. Epigenetic age was calculated as reported previously. The epigenetic clock is defined as a prediction method of age based on the DNAm levels of 353 CpGs. For the purpose of this study, we examined only old samples (age 72-77, n=7) and young (age 19-41, n=5).

12. Liu, Y., et al., *RNA-Seq identifies novel myocardial gene expression signatures of heart failure.* Genomics, 2015. **105**(2): p. 83-9.
--Samples were acquired from subjects at the MAGNet consortium. The RNA-Seq data were aligned to the hg19 reference genome using Tophat with default options, and transcripts were assembled with Cufflinks to compare normal versus ischemic samples. Genes differentially expressed (FDR adjusted p-value <0.05) between non-failure and ischemic hearts (n=6) were identified and applied to classify mRNA data of a larger cohort of 313 patients.

13. Simunovic, F., et al., *Evidence for gender-specific transcriptional profiles of nigral dopamine neurons in Parkinson disease.* PLoS One, 2010. **5**(1): p. e8856.
-- RNA was analyzed using laser microdissection of neurons in post-mortem brains from Parkinson compared to age-matched controls, in male and female groups. Data were analyzed using 3-way analysis of variance (ANOVA) and significance analysis of microarrays (SAM) procedure after removing a batch effect. The enrichment-based, pathway-level comparative computational analysis was largely done according to previously published protocols.
