# Supplementary material for: Global mapping of transcription factor motifs in human aging
Source: PLoS One. 2018 Jan 2;13(1):e0190457. doi: 10.1371/journal.pone.0190457 (PMC5749797; doi:10.1371/journal.pone.0190457)
Supplement: S2 File — (PDF) [file pone.0190457.s004.pdf]

Supplemental File S2: List of Up- and Down-regulated Genes Per Sample.

Top 75 up-regulated and bottom 75 down-regulated genes used in the transcription factor analysis. Table includes Refseq and corresponding gene name.

ID#	Sample	Up-Reg. Gene (Refseq)	Corresponding Gene Name	Down-Reg. Gene (Refseq)	Corresponding Gene Name
1	**Fibroblasts: Senescence/ Proliferation**	NM_000587	C7	NM_000057	BLM
		NM_000600	IL6	NM_000104	CYP1B1
		NM_000900	MGP	NM_000609	CXCL12
		NM_000963	PTGS2	NM_000946	PRIM1
		NM_001004439	ITGA11	NM_000956	PTGER2
		NM_001013398	IGFBP3	NM_001012410	SGO1
		NM_001013732	PTCHD4	NM_001039535	SKA1
		NM_001017992	ACTBL2	NM_001130688	HMGB2
		NM_001025077	CELF2	NM_001142556	HMMR
		NM_001033045	GPR155	NM_001145775	FKBP5
		NM_001077197	PDE11A	NM_001211	BUB1B
		NM_001122752	SERPINI1	NM_001717	BNC1
		NM_001123387	KRTAP2-1	NM_002417	MKI67
		NM_001135599	TGFB2	NM_002692	POLE2
		NM_001156474	CCDC81	NM_003318	TTK
		NM_001338	CXADR	NM_003510	HIST1H2AK
		NM_001759	CCND2	NM_003513	HIST1H2AB
		NM_001854	COL11A1	NM_003521	HIST1H2BM
		NM_001955	EDN1	NM_003522	HIST1H2BF
		NM_002019	FLT1	NM_003525	HIST1H2BI
		NM_002581	PAPPA	NM_003529	HIST1H3A
		NM_003059	SLC22A4	NM_003535	HIST1H3J
		NM_003220	TFAP2A	NM_003537	HIST1H3B
		NM_003326	TNFSF4	NM_003540	HIST1H4F
		NM_003480	MFAP5	NM_003544	HIST1H4B
		NM_004170	SLC1A1	NM_004100	EYA4
		NM_004415	DSP	NM_004456	EZH2
		NM_004572	PKP2	NM_004523	KIF11
		NM_005398	PPP1R3C	NM_004560	ROR2
		NM_005562	LAMC2	NM_004787	SLIT2
		NM_006074	TRIM22	NM_004900	APOBEC3B
		NM_006727	CDH10	NM_005045	RELN
		NM_014357	LCE2B	NM_005159	ACTC1
		NM_014391	ANKRD1	NM_005192	CDKN3
		NM_014399	TSPAN13	NM_005322	HIST1H1B
		NM_014840	NUAK1	NM_005325	HIST1H1A
		NM_014900	COBLL1	NM_005431	XRCC2
		NM_016591	GCNT4	NM_005573	LMNB1
		NM_018712	ELMOD1	NM_005691	ABCC9
		NM_018933	PCDHB13	NM_005733	KIF20A
		NM_018934	PCDHB14	NM_006439	MAB21L2
		NM_019120	PCDHB8	NM_012177	FBXO5
		NM_020796	SEMA6A	NM_012310	KIF4A
		NM_021101	CLDN1	NM_014264	PLK4
		NM_023037	FRY	NM_014729	TOX
		NM_025107	MYCT1	NM_014736	PCLAF
		NM_025214	CCDC68	NM_014783	ARHGAP11A
		NM_030955	ADAMTS12	NM_014875	KIF14
		NM_031246	PSG2	NM_015341	NCAPH

		NM_032812	PLXDC2	NM_015975	TAF9B
		NM_033184	KRTAP2-4	NM_016343	CENPF
		NM_052880	PIK3IP1	NM_016359	NUSAP1
		NM_054032	MRGPRX4	NM_017669	ERCC6L
		NM_057168	WNT16	NM_017770	ELOVL2
		NM_139248	LIPH	NM_018136	ASPM
		NM_139314	ANGPTL4	NM_018248	NEIL3
		NM_152703	SAMD9L	NM_018431	DOK5
		NM_153267	MAMDC2	NM_020242	KIF15
		NM_175056	ZPLD1	NM_020675	SPC25
		NM_175739	SERPINA9	NM_021052	HIST1H2AE
		NM_178428	LCE2A	NM_021062	HIST1H2BB
		NM_178429	LCE2C	NM_021066	HIST1H2AJ
		NM_181785	SLC46A3	NM_031942	CDCA7
		NM_203371	FIBIN	NM_032117	MND1
		NM_206966	C5orf46	NM_033084	FANCD2
		NM_207015	NAALADL2	NM_130398	EXO1
		NM_175739	SERPINA9	NM_145018	DDIAS
		NM_178429	LCE2C	NM_145061	SKA3
		NM_139314	ANGPTL4	NM_145697	NUF2
		NM_206966	C5orf46	NM_175065	HIST2H2AB
		NM_175056	ZPLD1	NM_182751	MCM10
		NM_001077197	PDE11A	NM_198947	FAM111B
		NM_014357	LCE2B	NM_202002	FOXM1
		NM_005562	LAMC2	NM_002692	POLE2
		NM_006727	CDH10	NM_018136	ASPM
2	**Fibroblasts: Senescence/ Quiescence I**	NM_000192	TBX5	NM_000808	GABRA3
		NM_001003818	TRIM6	NM_001001924	MTUS1
		NM_001018005	TPM1	NM_001005336	DNM1
		NM_001081754	ELN	NM_001005473	PLCXD3
		NM_001098520	HTATIP2	NM_001020658	PUM1
		NM_001099691	TGFA	NM_001077198	ATG9A
		NM_001122670	PHKA1	NM_001081677	SCN3A
		NM_001125	ADPRH	NM_001082618	CD33
		NM_001128205	SULF1	NM_001098500	KIAA1217
		NM_001136472	LITAF	NM_001113397	ZNF385B
		NM_001136473	LITAF	NM_001123067	MAPT
		NM_001143963	L1CAM	NM_001128224	TMCC1
		NM_001145771	ADGRG1	NM_001143886	PPP1R12A
		NM_001161560	TNIK	NM_001145145	SLC1A5
		NM_001161561	TNIK	NM_001166387	MAGEA12
		NM_001163809	WDR81	NM_001172173	CSRNP3
		NM_001178108	ZNF185	NM_001172832	ZNF300
		NM_001178110	ZNF185	NM_001178140	TFDP2
		NM_001198644	TRIM6	NM_001195000	MR1
		NM_001199763	PTPRN	NM_001198764	CD302
		NM_001242946	EPDR1	NM_001203251	MAPT
		NM_001256155	ARMCX4	NM_001203252	MAPT
		NM_001258323	TP53I11	NM_001204404	ANK3
		NM_001270764	CHST15	NM_001206901	TAX1BP1
		NM_001289024	KIF6	NM_001256255	ADGRE1
		NM_001297592	SEL1L3	NM_001256627	BRSK2
		NM_001301210	MYEF2	NM_001258379	SLC4A7
		NM_001301244	TPM1	NM_001270976	IST1
		NM_001303108	TMEM246	NM_001272065	SHISA5
		NM_001309192	EPHB2	NM_001276	CHI3L1

		NM_001310319	MFGE8	NM_001278580	ACVR2A
		NM_001311	CRIP1	NM_001286367	NOP9
		NM_001490	GCNT1	NM_001289160	MICB
		NM_001546	ID4	NM_001290043	TAP2
		NM_001996	FBLN1	NM_001301289	TPM1
		NM_001999	FBN2	NM_001303145	PCDHB6
		NM_002487	NDN	NM_001308278	SLC14A1
		NM_003012	SFRP1	NM_001310213	MR1
		NM_003236	TGFA	NM_001317188	BRINP3
		NM_003266	TLR4	NM_001317222	CDH10
		NM_003500	ACOX2	NM_001317224	CDH10
		NM_003781	B3GALNT1	NM_001317227	CDH12
		NM_004350	RUNX3	NM_001321108	SLC4A7
		NM_004415	DSP	NM_001330344	TPM1
		NM_004442	EPHB2	NM_001330578	ATP7B
		NM_004538	NAP1L3	NM_001330989	RABGAP1L
		NM_004696	SLC16A4	NM_001449	FHL1
		NM_005103	FEZ1	NM_001772	CD33
		NM_005514	HLA-B	NM_003957	BRSK2
		NM_005547	IVL	NM_005014	OMD
		NM_005864	EFS	NM_005362	MAGEA3
		NM_006486	FBLN1	NM_005363	MAGEA6
		NM_007036	ESM1	NM_005367	MAGEA12
		NM_014310	RASD2	NM_005619	RTN2
		NM_014783	ARHGAP11A	NM_005931	MICB
		NM_015028	TNIK	NM_006727	CDH10
		NM_015170	SULF1	NM_006922	SCN3A
		NM_017549	EPDR1	NM_012268	PLD3
		NM_018196	TMLHE	NM_016279	CDH9
		NM_018649	H2AFY2	NM_016834	MAPT
		NM_020465	NDRG4	NM_016841	MAPT
		NM_021738	SVIL	NM_018939	PCDHB6
		NM_024003	L1CAM	NM_018940	PCDHB7
		NM_024017	HOXB9	NM_031372	HNRNPDL
		NM_024642	GALNT12	NM_032229	SLITRK6
		NM_025015	HSPA12A	NM_033140	CALD1
		NM_032342	TMEM246	NM_033355	CASP8
		NM_134268	CYGB	NM_080630	COL11A1
		NM_138455	CTHRC1	NM_145322	OSBPL3
		NM_144497	AKAP12	NM_170706	NMNAT2
		NM_148957	TNFRSF19	NM_172216	CAMKK2
		NM_152380	TBX15	NM_173627	ENDOV
		NM_174938	FRMD3	NM_177444	PPFIBP1
		NM_182904	P4HA3	NM_194250	ZNF804A
		NM_183376	ARRDC4	NM_199051	BRINP3
		NM_198148	CPXM2		
		NM_199357	ARHGAP11A		
		NM_201524	ADGRG1		
		NM_201526	ISLR		
		NM_206925	CA12		
3	**Fibroblasts: Senescence/ Quiescence II**	NM_000118	ENG	NM_000216	ANOS1
		NM_000196	HSD11B2	NM_000444	PHEX
		NM_000327	ROM1	NM_000525	KCNJ11
		NM_000428	LTBP2	NM_000586	IL2
		NM_000595	LTA	NM_000865	HTR1E
		NM_000805	GAST	NM_000963	PTGS2

	NM_000834	GRIN2B	NM_001007544	C1orf186
	NM_001004339	ZYG11A	NM_001022	RPS19
	NM_001008404	C14orf180	NM_001086	AADAC
	NM_001018	RPS15	NM_001117	ADCYAP1
	NM_001029	RPS26	NM_001242	CD27
	NM_001093	ACACB	NM_001321	CSRP2
	NM_001185	AZGP1	NM_001496	GFRA3
	NM_001495	GFRA2	NM_001546	ID4
	NM_001549	IFIT3	NM_001899	CST4
	NM_001935	DPP4	NM_002294	LAMP2
	NM_002220	ITPKA	NM_002517	NPAS1
	NM_002461	MVD	NM_002638	PI3
	NM_002727	SRGN	NM_002660	PLCG1
	NM_002808	PSMD2	NM_002771	PRSS3
	NM_002874	RAD23B	NM_002818	PSME2
	NM_002993	CXCL6	NM_003013	SFRP2
	NM_003248	THBS4	NM_003286	TOP1
	NM_003254	TIMP1	NM_003650	CST7
	NM_003598	TEAD2	NM_003710	SPINT1
	NM_003868	FGF16	NM_003986	BBOX1
	NM_003881	WISP2	NM_004036	ADCY3
	NM_004164	RBP2	NM_004270	MED7
	NM_004415	DSP	NM_004357	CD151
	NM_004547	NDUFB4	NM_004450	ERH
	NM_004967	IBSP	NM_004494	HDGF
	NM_005143	HP	NM_004724	ZW10
	NM_005194	CEBPB	NM_004895	NLRP3
	NM_005268	GJB5	NM_004924	ACTN4
	NM_005433	YES1	NM_005197	FOXN3
	NM_005512	LRRC32	NM_005329	HAS3
	NM_005602	CLDN11	NM_005602	CLDN11
	NM_005714	KCNK7	NM_005913	MC5R
	NM_006242	PPP1R3D	NM_006061	CRISP3
	NM_006549	CAMKK2	NM_006259	PRKG2
	NM_006941	SOX10	NM_006738	AKAP13
	NM_013450	BAZ2B	NM_006833	COPS6
	NM_014363	SACS	NM_006930	SKP1
	NM_014990	RALGAPA1	NM_007375	TARDBP
	NM_018302	C4orf19	NM_014353	RAB26
	NM_018667	SMPD3	NM_014371	AKAP8L
	NM_020932	MAGEE1	NM_014520	MYBBP1A
	NM_021615	CHST6	NM_014926	SLITRK3
	NM_021808	GALNT9	NM_015259	ICOSLG
	NM_022154	SLC39A8	NM_015425	POLR1A
	NM_022768	RBM15	NM_015564	LRRTM2
	NM_022826	MARCH7	NM_015894	STMN3
	NM_022828	YTHDC2	NM_015958	DPH5
	NM_024298	MBOAT7	NM_017416	IL1RAPL2
	NM_024725	CCDC82	NM_017888	ACSM5
	NM_032287	RTL6	NM_018990	SASH3
	NM_032487	ACTRT3	NM_019034	RHOF
	NM_032590	KDM2B	NM_022036	GPRC5C
	NM_033118	MYLK2	NM_024298	MBOAT7
	NM_052862	RCSD1	NM_024508	ZBED2
	NM_052882	ZIM3	NM_024527	ABHD8
	NM_080593	HIST1H2BK	NM_024795	TM4SF20

		NM_138413<br>NM_139163<br>NM_139319<br>NM_144575<br>NM_145740<br>NM_152250<br>NM_152605<br>NM_152898<br>NM_153217<br>NM_153703<br>NM_170745<br>NM_173567<br>NM_173828	HOGA1<br>ALS2CR12<br>SLC17A8<br>CAPN13<br>GSTA1<br>DEFB105A<br>ZNF781<br>FERD3L<br>TMEM174<br>PODN<br>HIST1H2AA<br>EPHX4<br>RELL2	NM_025210<br>NM_031422<br>NM_031862<br>NM_032369<br>NM_033207<br>NM_052942<br>NM_138288<br>NM_138340<br>NM_138569<br>NM_145174<br>NM_174912<br>NM_174913<br>NM_181844	PPP1R2P9<br>CHST9<br>NBR1<br>HVCN1<br>OPALIN<br>GBP5<br>SPTSSA<br>ABHD3<br>MLIP<br>DNAJB7<br>FAAH2<br>NOP9<br>BCL6B
4	**Fibroblasts: ERiQ**	NM_000090<br>NM_000527<br>NM_000859<br>NM_000903<br>NM_001098816<br>NM_001124<br>NM_001548<br>NM_001850<br>NM_002048<br>NM_002130<br>NM_002185<br>NM_002340<br>NM_002546<br>NM_002780<br>NM_003246<br>NM_003247<br>NM_003485<br>NM_003500<br>NM_003713<br>NM_003739<br>NM_003873<br>NM_004104<br>NM_004462<br>NM_004508<br>NM_005063<br>NM_005542<br>NM_005602<br>NM_005891<br>NM_006009<br>NM_006195<br>NM_006206<br>NM_006675<br>NM_012093<br>NM_012098<br>NM_012242<br>NM_013282<br>NM_013409<br>NM_014220<br>NM_014573<br>NM_014762<br>NM_014862<br>NM_015419	COL3A1<br>LDLR<br>HMGCR<br>NQO1<br>TENM4<br>ADM<br>IFIT1<br>COL8A1<br>GAS1<br>HMGCS1<br>IL7R<br>LSS<br>TNFRSF11B<br>PSG4<br>THBS1<br>THBS2<br>GPR68<br>ACOX2<br>PLPP3<br>AKR1C3<br>NRP1<br>FASN<br>FDFT1<br>IDI1<br>SCD<br>INSIG1<br>CLDN11<br>ACAT2<br>TUBA1A<br>PBX3<br>PDGFRA<br>TSPAN9<br>AK5<br>ANGPTL2<br>DKK1<br>UHRF1<br>FST<br>TM4SF1<br>TMEM97<br>DHCR24<br>ARNT2<br>MXRA5	NM_000407<br>NM_000600<br>NM_001012974<br>NM_001013251<br>NM_001017425<br>NM_001025366<br>NM_001146<br>NM_001717<br>NM_002056<br>NM_002201<br>NM_002220<br>NM_002514<br>NM_003324<br>NM_003376<br>NM_003486<br>NM_004083<br>NM_004183<br>NM_004675<br>NM_004733<br>NM_004793<br>NM_004794<br>NM_004823<br>NM_004864<br>NM_004933<br>NM_005098<br>NM_005384<br>NM_005851<br>NM_006134<br>NM_006636<br>NM_006934<br>NM_007076<br>NM_012162<br>NM_012248<br>NM_012328<br>NM_013388<br>NM_014445<br>NM_014637<br>NM_015359<br>NM_015525<br>NM_015599<br>NM_015641<br>NM_016270	GP1BB<br>IL6<br>LRRC73<br>SLC3A2<br>KCNK2<br>VEGFA<br>ANGPT1<br>BNC1<br>GFPT1<br>ISG20<br>ITPKA<br>NOV<br>TULP3<br>VEGFA<br>SLC7A5<br>DDIT3<br>BEST1<br>DIRAS3<br>SLC33A1<br>LONP1<br>RAB33A<br>KCNK6<br>GDF15<br>CDH15<br>MSC<br>NFIL3<br>CDK2AP2<br>TMEM50B<br>MTHFD2<br>SLC6A9<br>FICD<br>FBXL6<br>SEPHS2<br>DNAJB9<br>PREB<br>SERP1<br>MTFR1<br>SLC39A14<br>IBTK<br>PGM3<br>TES<br>KLF2

		NM_015441	OLFML2B	NM_016498	MTFP1
		NM_016095	GINS2	NM_017786	SYBU
		NM_016613	FAM198B	NM_017947	MOCOS
		NM_018153	ANTXR1	NM_018177	N4BP2
		NM_018689	CEMIP	NM_018420	SLC22A15
		NM_019035	PCDH18	NM_019089	HES2
		NM_020351	COL8A1	NM_019096	GTPBP2
		NM_020954	RNF213	NM_020127	TUFT1
		NM_021111	RECK	NM_021101	CLDN1
		NM_022469	GREM2	NM_021147	CCNO
		NM_025081	NYNRIN	NM_021158	TRIB3
		NM_030966	KRTAP1-3	NM_021237	SELENOK
		NM_030967	KRTAP1-1	NM_021958	HLX
		NM_031243	HNRNPA2B1	NM_022082	SLC17A9
		NM_031302	GLT8D2	NM_022117	TSPYL2
		NM_032291	SGIP1	NM_024111	CHAC1
		NM_032623	MGARP	NM_024555	FBXL6
		NM_053064	GNG2	NM_030674	SLC38A1
		NM_080927	DCBLD2	NM_031479	INHBE
		NM_133468	BMPER	NM_032239	LARP1B
		NM_145260	OSR1	NM_032431	SYVN1
		NM_145693	LPIN1	NM_032683	MPV17L2
		NM_153000	APCDD1	NM_033103	RHPN2
		NM_173485	TSHZ2	NM_033452	TRIM47
		NM_177403	RAB7B	NM_139314	ANGPTL4
		NM_177964	LYPD6B	NM_152270	SLFN11
		NM_198336	INSIG1	NM_152331	ACOT4
		NM_199168	CXCL12	NM_152829	TES
		NM_203329	CD59	NM_152995	NFXL1
		NM_206929	SYTL2	NM_153742	CTH
		NM_206930	SYTL2	NM_177968	PPM1B
		NM_207645	C11orf87	NM_178815	ARL5B
		NM_001076552	ACSS2	NM_182491	ZFAND2A
				NM_201525	ADGRG1
				NM_203472	SELENOS
5	**Fibroblasts: Cross-Section**	NM_000032	ALAS2	NM_000640	IL13RA2
		NM_000118	ENG	NM_001000	RPL39
		NM_000672	ADH6	NM_001662	ARF5
		NM_000728	CALCB	NM_001681	ATP2A2
		NM_000802	FOLR1	NM_001688	ATP5F1
		NM_001276	CHI3L1	NM_001839	CNN3
		NM_001298	CNGA3	NM_001928	CFD
		NM_001937	DPT	NM_001961	EEF2
		NM_001949	E2F3	NM_001970	EIF5A
		NM_002089	CXCL2	NM_001999	FBN2
		NM_002221	ITPKB	NM_002889	RARRES2
		NM_002499	NEO1	NM_002998	SDC2
		NM_002575	SERPINB2	NM_003093	SNRPC
		NM_002864	PZP	NM_003286	TOP1
		NM_003007	SEMG1	NM_003730	RNASET2
		NM_003034	ST8SIA1	NM_003796	URI1
		NM_003881	WISP2	NM_004104	FASN
		NM_003980	MAP7	NM_004199	P4HA2
		NM_004057	S100G	NM_004265	FADS2
		NM_004070	CLCNKA	NM_004467	FGL1
		NM_004598	SPOCK1	NM_004572	PKP2

	NM_004673	ANGPTL1	NM_004780	TCEAL1
	NM_004724	ZW10	NM_004789	LHX2
	NM_004898	CLOCK	NM_004861	GAL3ST1
	NM_005127	CLEC2B	NM_005086	SSPN
	NM_005549	KCNA10	NM_005176	ATP5G2
	NM_005860	FSTL3	NM_005254	GABPB1
	NM_006059	LAMC3	NM_005255	GAK
	NM_006352	ZBTB18	NM_005398	PPP1R3C
	NM_006477	RASL10A	NM_005572	LMNA
	NM_006573	TNFSF13B	NM_005801	EIF1
	NM_007118	TRIO	NM_005985	SNAI1
	NM_012098	ANGPTL2	NM_006471	MYL12A
	NM_012198	GCA	NM_007207	DUSP10
	NM_013330	NME7	NM_012080	PUDP
	NM_014659	PPIP5K1	NM_012152	LPAR3
	NM_014916	LMTK2	NM_013386	SLC25A24
	NM_015662	IFT172	NM_014164	FXYD5
	NM_018062	FANCL	NM_014279	OLFM1
	NM_018154	ASF1B	NM_014474	SMPDL3B
	NM_018254	RCOR3	NM_014899	RHOBTB3
	NM_020414	DDX24	NM_015144	ZCCHC14
	NM_021647	MFAP3L	NM_016206	VGLL3
	NM_024298	MBOAT7	NM_016522	NTM
	NM_024551	ADIPOR2	NM_018035	DMAC2
	NM_024669	ANKRD55	NM_018686	CMAS
	NM_024942	C10orf88	NM_018955	UBB
	NM_031950	FGFBP2	NM_020381	PDSS2
	NM_032250	ANKRD20A1	NM_020461	TUBGCP6
	NM_032549	IMMP2L	NM_020645	NRIP3
	NM_032590	KDM2B	NM_020754	ARHGAP31
	NM_032752	ZNF496	NM_021061	ZNF250
	NM_032792	ZBTB45	NM_021827	CCDC81
	NM_033124	CCDC65	NM_021955	GNGT1
	NM_080874	ASB5	NM_021973	HAND2
	NM_139164	STARD4	NM_021979	HSPA2
	NM_144603	NOXO1	NM_022047	DEF6
	NM_144722	SPEF2	NM_022805	SNRPN
	NM_147156	SGMS1	NM_024603	BEND5
	NM_147193	GLIS1	NM_024645	ZMAT4
	NM_153216	POU5F2	NM_030799	YIPF5
	NM_170745	HIST1H2AA	NM_030915	LBH
	NM_175067	TAAR6	NM_032026	TATDN1
	NM_182592	YIPF7	NM_080429	AQP10
	NM_194447	CLEC4A	NM_080748	ROMO1
	NM_198461	LONRF2	NM_145239	PRRT2
	NM_199254	TPTE2	NM_152608	SDE2
	NM_203412	UBL4B	NM_152892	LRWD1
	NM_207517	ADAMTSL3	NM_175709	CBX7
	NM_212551	LYSMD1	NM_181655	C17orf58
	NM_153031	SATB2-AS1	NM_198265	SPO11
	NM_173661	LINC00955	NM_203377	MB
	NM_183414	UBE3B	NM_212559	XKRX
	NM_002499	NEO1	NM_018686	CMAS
	NM_006573	TNFSF13B	NM_004789	LHX2

6	**Progeria Cells**	NM_004101	F2RL2	NM_001256099	CTHRC1
		NM_004482	GALNT3	NM_001199723	CRABP2
		NM_002982	CCL2	NM_001271166	PTGS1
		NM_007257	PNMA2	NM_012244	SLC7A8
		NM_000104	CYP1B1	NM_033642	FGF13
		NM_001142316	LMO2	NM_002422	MMP3
		NM_001100167	MRVI1	NM_002133	HMOX1
		NM_000214	JAG1	NM_001452	FOXF2
		NM_001185056	CLDN11	NM_053064	GNG2
		NM_002514	NOV	NM_001160001	NRG1
		NM_001257135	LIF	NM_000493	COL10A1
		NM_005567	LGALS3BP	NM_000640	IL13RA2
		NM_006950	SYN1	NM_001047160	NET1
		NM_004006	DMD	NM_001165924	EMX2
		NM_002977	SCN9A	NM_181351	NCAM1
		NM_021021	SNTB1	NM_012307	EPB41L3
		NM_022475	HHIP	NM_001006946	SDC1
		NM_002852	PTX3	NM_172113	EYA2
		NM_177524	MEST	NM_001206650	PSG7
		NM_003004	SECTM1	NM_172244	SGCD
		NM_001130081	PLD1	NM_005328	HAS2
		NM_000958	PTGER4	NM_003222	TFAP2C
		NM_031308	EPPK1	NM_001185055	ADD2
		NM_004334	BST1	NM_014310	RASD2
		NM_001105250	NRXN3	NM_001252197	IRX5
		NR_026881	PRG1	NM_006208	ENPP1
		NM_032961	PCDH10	NM_002160	TNC
		NM_021229	NTN4	NM_001831	CLU
		NM_000900	MGP	NM_024337	IRX1
		NM_007361	NID2	NM_001294346	NINJ2
		NM_014331	SLC7A11	NM_001794	CDH4
		NM_000495	COL4A5	NM_021724	NR1D1
		NM_182472	EPHA5	NM_000095	COMP
		NM_033035	TSLP	NM_022898	BCL11B
		NM_005532	IFI27	NM_139072	DNER
		NM_000224	KRT18	NM_031908	C1QTNF2
		NM_005924	MEOX2	NM_013230	CD24
		NM_001134367	SLC6A6	NM_005202	COL8A2
		NM_006207	PDGFRL	NM_001288585	LACTB
		NM_001032281	TFPI	NM_001282611	OLFM1
		NM_004473	FOXE1	NM_001937	DPT
		NM_002575	SERPINB2	NM_006042	HS3ST3A1
		NM_001814	CTSC	NM_002527	NTF3
		NM_001145206	KIAA1671	NM_080717	TBX5
		NM_001311313	F2R	NM_024812	BAALC
		NM_145640	APOL3	NM_002048	GAS1
		NM_000955	PTGER1	NM_033517	SHANK3
		NM_024420	PLA2G4A	NM_201567	CDC25A
		NM_001845	COL4A1	NM_175081	P2RX5
		NM_006895	HNMT	NM_001130675	CLGN
		NM_005257	GATA6	NM_001127892	SALL1
		NM_001295	CCR1	NM_003786	ABCC3
		NM_005556	KRT7	NM_001204961	PBX1
		NM_013409	FST	NM_182826	SCARA3
		NM_001244134	MAP3K8	NM_001150	ANPEP
		NM_001282778	RGS7	NM_001256139	CAPG

		NM_173054	RELN	NM_198217	ING1
		NM_005711	EDIL3	NM_002250	KCNN4
		NM_004932	CDH6	NM_001271893	TWIST2
		NM_005763	AASS	NM_001271003	TFPI2
		NM_003247	THBS2	NM_002448	MSX1
		NM_014729	TOX	NM_003155	STC1
		NM_021991	JUP	NM_000956	PTGER2
		NM_017784	OSBPL10	NM_001049	SSTR1
		NM_002935	RNASE3	NM_001099294	KIAA1644
		NM_022341	PDF	NM_000049	ASPA
		NM_001305004	PLP1	NM_005985	SNAI1
		NM_007315	STAT1	NM_004978	KCNC4
		NM_005560	LAMA5	NM_001256339	NKX3-1
		NM_001300994	TPD52L1	NM_145173	DIRAS1
		NM_001318388	TP53I11	NM_001204086	NBL1
		NM_005098	MSC	NM_001184	ATR
		NM_001278914	ELN	NM_014021	SSX2IP
		NM_005204	MAP3K8	NM_201634	TCF7
		NM_005711	EDIL3	NM_002252	KCNS3
7	**Progeria Experimental Model**	NM_000150	FUT6	NM_000039	APOA1
		NM_000575	IL1A	NM_000090	COL3A1
		NM_000576	IL1B	NM_000204	CFI
		NM_000584	CXCL8	NM_000554	CRX
		NM_000600	IL6	NM_000557	GDF5
		NM_000735	CGA	NM_000599	IGFBP5
		NM_000758	CSF2	NM_000670	ADH4
		NM_000915	OXT	NM_000961	PTGIS
		NM_000953	PTGDR	NM_001007544	C1orf186
		NM_000963	PTGS2	NM_001327	CTAG1B
		NM_001165	BIRC3	NM_001461	FMO5
		NM_001364	DLG2	NM_001822	CHN1
		NM_001432	EREG	NM_001854	COL11A1
		NM_001700	AZU1	NM_001937	DPT
		NM_001766	CD1D	NM_002193	INHBB
		NM_001888	CRYM	NM_002362	MAGEA4
		NM_001946	DUSP6	NM_002404	MFAP4
		NM_002089	CXCL2	NM_002550	OR3A1
		NM_002090	CXCL3	NM_003278	CLEC3B
		NM_002153	HSD17B2	NM_003287	TPD52L1
		NM_002163	IRF8	NM_003296	CRISP2
		NM_002262	KLRD1	NM_003739	AKR1C3
		NM_002348	LY9	NM_003882	WISP1
		NM_002421	MMP1	NM_003956	CH25H
		NM_002638	PI3	NM_004428	EFNA1
		NM_002928	RGS16	NM_004438	EPHA4
		NM_002974	SERPINB4	NM_004525	LRP2
		NM_003122	SPINK1	NM_004669	CLIC3
		NM_003245	TGM3	NM_004753	DHRS3
		NM_003483	HMGA2	NM_004934	CDH18
		NM_004490	GRB14	NM_004962	GDF10
		NM_004644	AP3B2	NM_005139	ANXA3
		NM_004675	DIRAS3	NM_005259	MSTN
		NM_005073	SLC15A1	NM_005602	CLDN11
		NM_005397	PODXL	NM_005622	ACSM3
		NM_005408	CCL13	NM_005810	KLRG1
		NM_005524	HES1	NM_005822	RCAN2

		NM_005842	SPRY2	NM_005912	MC4R
		NM_005951	MT1H	NM_006774	INMT
		NM_006290	TNFAIP3	NM_006863	LILRA1
		NM_006350	FST	NM_006872	GTF2A1L
		NM_006762	LAPTM5	NM_006974	ZNF33A
		NM_006823	PKIA	NM_012113	CA14
		NM_006846	SPINK5	NM_014460	CSDC2
		NM_007036	ESM1	NM_014476	PDLIM3
		NM_007115	TNFAIP6	NM_015852	ZNF117
		NM_014143	CD274	NM_017833	DNAJC28
		NM_014452	TNFRSF21	NM_018099	FAR2
		NM_017791	FLVCR2	NM_018280	PRR34
		NM_017826	SOHLH2	NM_018640	LMO3
		NM_018354	TMEM74B	NM_019596	C21orf62
		NM_020997	LEFTY1	NM_021192	HOXD11
		NM_021938	CELF5	NM_024560	ACSS3
		NM_022475	HHIP	NM_025151	RAB11FIP1
		NM_024768	EFCC1	NM_030965	ST6GALNAC5
		NM_031882	PCDHAC1	NM_032526	NT5C1A
		NM_032505	KBTBD8	NM_032551	KISS1R
		NM_032793	MFSD2A	NM_033337	CAV3
		NM_032865	TNS4	NM_080662	PEX11G
		NM_138966	NETO1	NM_080751	TMC2
		NM_152638	CCER1	NM_139167	SGCZ
		NM_152677	ZSCAN4	NM_145234	CHRDL1
		NM_153456	HS6ST3	NM_152366	KLHDC9
		NM_174959	SVOPL	NM_152467	KLHL10
		NM_176810	NLRP13	NM_172004	CLECL1
		NM_176875	CCKBR	NM_173086	KRT6C
		NM_178428	LCE2A	NM_173554	C10orf107
		NM_181079	IL21R	NM_176816	CCDC125
		NM_181353	ID1	NM_181332	NLGN4X
		NM_181847	AMIGO2	NM_207338	LCTL
		NM_002071	GNAL	NM_021208	C9orf27
		NM_004024	ATF3	NM_194303	JAKMIP3
		NM_025002	C6orf208	NM_173507	C1orf127
		NM_030878	CYP2C8	NM_181797	KCNQ1
		NM_145033	LINC00161	NM_182494	CALHM3
8	**Adipose (Subcut.)**	NM_152501	PYHIN1	NM_033448	KRT71
		NM_199037	SCN1B	NM_001006658	CR2
		NM_001324201	EDA2R	NM_001136007	FXYD3
		NM_001301012	EYA4	NM_177972	TUB
		NM_001099692	EIF5AL1	NM_001242498	MIF4GD
		NM_001453	FOXC1	NM_001139443	BEST1
		NM_001143976	WEE1	NM_017999	RNF31
		NM_003198	TCEB3	NM_006178	NSF
		NM_001144857	PLEKHG6	NM_000655	SELL
		NM_001318539	BEND6	NM_018094	GSPT2
		NM_002056	GFPT1	NM_014689	DOCK10
		NM_153223	CEP120	NM_014245	RNF7
		NM_002228	JUN	NM_022901	LRRC19
		NM_015177	DTX4	NM_003965	CCRL2
		NM_021009	UBC	NM_001320531	SLC9C1
		NM_001243612	LMO3	NM_001032393	HNRNPH2
		NM_001077397	IRF2BP2	NM_014898	ZFP30
		NM_199340	LRRC37A3	NM_001253357	TIE1

	NM_003437	ZNF136	NM_033257	DGCR6L
	NM_001689	ATP5G3	NM_025195	TRIB1
	NM_006195	PBX3	NM_000353	TAT
	NM_016239	MYO15A	NM_015155	LARP4B
	NM_145053	UBQLNL	NM_006200	PCSK5
	NM_001197317	CLEC2D	NM_020637	FGF22
	NM_001303110	COL4A1	NM_001818	AKR1C4
	NM_005020	PDE1C	NM_173204	IL37
	NM_000107	DDB2	NM_015464	SOSTDC1
	NM_030810	TXNDC5	NM_001004313	TMEM220
	NM_006111	ACAA2	NM_004398	DDX10
	NM_022470	ZMAT3	NM_001320942	IREB2
	NM_018433	KDM3A	NM_152635	OIT3
	NM_001195082	TEX22	NM_001308081	DTWD2
	NM_001975	ENO2	NM_033114	ZCRB1
	NM_138408	GTF3C6	NM_030961	TRIM56
	NM_033386	MICALL1	NM_001320871	SLC25A35
	NM_001036	RYR3	NM_014688	USP6NL
	NM_001846	COL4A2	NM_005408	CCL13
	NM_015553	IPCEF1	NM_001287527	CLIP4
	NM_152909	ZNF548	NM_001317851	HEMK1
	NM_022051	EGLN1	NM_138715	MSR1
	NM_001251902	TNK1	NM_001102470	ADH6
	NM_001079534	CPEB1	NM_001423	EMP1
	NM_001300836	PLEKHJ1	NM_006948	HSPA13
	NM_000043	FAS	NM_181491	MED22
	NM_000050	ASS1	NM_001193530	CCT6B
	NM_004957	FPGS	NM_001002255	SUMO4
	NM_001322802	NNAT	NM_001135037	MFAP3
	NM_001080849	DNLZ	NM_144569	SPOCD1
	NM_170697	ALDH1A2	NM_001144999	ITGAV
	NM_004609	TCF15	NM_001080426	DUSP27
	NM_003268	TLR5	NM_006680	ME3
	NM_020251	ARRB1	NM_001633	AMBP
	NM_003451	ZNF177	NM_001204856	SCNM1
	NM_001556	IKBKB	NM_006788	RALBP1
	NM_030805	LMAN2L	NM_005891	ACAT2
	NM_001014436	DBNL	NM_177989	ACTL6A
	NM_001442	FABP4	NM_001292003	KLHL8
	NM_138991	BACE2	NM_005969	NAP1L4
	NM_001304444	ALAS1	NM_181272	CMTM1
	NM_001142576	IMPDH1	NM_004227	CYTH3
	NM_001316307	RAC3	NM_001167574	UPK3A
	NM_001897	CSPG4	NM_003087	SNCG
	NM_001167820	BLCAP	NM_001286219	TMEM132B
	NM_145756	ZNF396	NM_001286167	FANCA
	NM_001538	HSF4	NM_001166400	MAGEA8
	NM_198717	PTGER3	NM_001318203	VTI1A
	NM_198443	NRN1L	NM_003671	CDC14B
	NM_015714	G0S2	NM_001171653	ZEB2
	NM_013451	MYOF	NM_003380	VIM
	NM_001001523	RORC	NM_198682	GYPE
	NM_006830	UQCR11	NM_178128	FADS6
	NM_001331027	HADH	NM_001011720	XKR9
	NM_012394	PFDN2	NM_001244764	TNIP3
	NM_001319137	CTSH	NM_014239	EIF2B2

		NM_014330	PPP1R15A	NM_001142504	STARD8
		NM_001193644	PEX19	NM_001145971	RDH13
		NM_001127716	ETFA	NM_003721	RFXANK
		NM_002616	PER1	NM_015306	USP24
		NM_004998	MYO1E	NM_001330188	TBC1D30
		NM_004628	XPC		
9	**Artery (Tibia)**	NM_001167940	CADPS2	NM_173533	TDRD5
		NM_020815	PCDH10	NM_024769	CLMP
		NM_001278478	RUNX2	NM_001693	ATP6V1B2
		NM_004720	LPAR2	NM_002095	GTF2E2
		NM_178026	GGT7	NM_001166266	LTBP1
		NM_001324201	EDA2R	NM_002114	HIVEP1
		NM_001199573	TRIM22	NM_001164796	SLC25A26
		NM_001017425	KCNK2	NM_001306153	CCT5
		NM_001318372	PCDH9	NM_001004311	FIGLA
		NM_024676	SH3D21	NM_145863	ASB3
		NM_148962	OXER1	NM_021098	CACNA1H
		NM_080630	COL11A1	NM_172071	RC3H1
		NM_001081677	SCN3A	NM_012284	KCNH3
		NM_001168364	KRTCAP3	NM_001204063	CHURC1
		NM_001992	F2R	NM_031422	CHST9
		NM_033449	FCHSD1	NM_201441	TEAD4
		NM_018969	GPR173	NM_001318050	SDR16C5
		NM_002702	POU6F1	NM_001134851	TCF7
		NM_001144027	NDOR1	NM_002307	LGALS7
		NM_001320155	CCNL2	NM_018931	PCDHB11
		NM_080680	COL11A2	NM_001040100	SPTSSB
		NM_001080410	FBXO41	NM_001164373	GPN3
		NM_033200	LMF2	NM_001163547	SYNRG
		NM_001261	CDK9	NM_002310	LIFR
		NM_001258021	GRIA1	NM_020645	NRIP3
		NM_001243612	LMO3	NM_001130438	SPTAN1
		NM_006663	PPP1R13L	NM_003748	ALDH4A1
		NM_022772	EPS8L2	NM_000968	RPL4
		NM_000889	ITGB7	NM_012121	CDC42EP4
		NM_153221	CILP2	NM_001286522	PARP11
		NM_003467	CXCR4	NM_018257	PCMTD2
		NM_052958	C8orf34	NM_033315	RASL10B
		NM_001278736	CCL5	NM_022828	YTHDC2
		NM_001318852	MAPK8IP3	NM_001010922	BCL2L15
		NM_178129	P2RY8	NM_003728	UNC5C
		NM_001198779	CUL2	NM_033540	MFN1
		NM_014922	NLRP1	NM_181656	C17orf58
		NM_006019	TCIRG1	NM_183006	DLGAP4
		NM_022470	ZMAT3	NM_173664	ARL10
		NM_178863	KCTD13	NM_016932	SIX2
		NM_001321086	NPAS1	NM_001013734	RFPL4B
		NM_003089	SNRNP70	NM_005787	ALG3
		NM_012407	PICK1	NM_032826	SLC35B4
		NM_177939	P4HTM	NM_203433	PSMG1
		NM_001321229	HDAC6	NM_152653	UBE2E2
		NM_001178129	SEMA3E	NM_004085	TIMM8A
		NM_001184782	CXorf57	NM_001039844	ACBD7
		NM_016622	MRPL35	NM_001128215	LIPM
		NM_006231	POLE	NM_000678	ADRA1D
		NM_001135	ACAN	NM_002014	FKBP4

		NM_001321387	PTN	NM_001286373	TOMM40L
		NM_020226	PRDM8	NM_006828	ASCC3
		NM_007158	CSDE1	NM_001317818	SLC10A7
		NM_058195	CDKN2A	NM_001551	IGBP1
		NM_182895	SCARF2	NM_018061	PRPF38B
		NM_001003927	EVI2A	NM_153027	FAM218A
		NM_024430	PSTPIP2	NM_017880	C2orf42
		NM_145798	OSBPL7	NM_001286478	EIF3C
		NM_001258327	SFI1	NM_030571	NDFIP1
		NM_145263	SPATA18	NM_001042784	CCDC158
		NM_030648	SETD7	NM_024533	CHST5
		NM_025082	CENPT	NM_001320140	RPL6
		NM_000292	PHKA2	NM_001303275	ACLY
		NM_001100119	XRCC3	NM_152557	ZNF746
		NM_001493	GDI1	NM_001004488	OR2A25
		NM_001174122	ZFYVE27	NM_177400	NKX6-2
		NM_001197127	IRF3	NM_006385	ZNF211
		NM_001017981	RNF215	NM_015983	UBE2D4
		NM_001145873	CD8A	NM_003036	SKI
		NM_001184721	GYG1	NM_001286134	RIC8A
		NM_006680	ME3	NM_174889	NDUFAF2
		NM_001256494	MAN2C1	NM_002795	PSMB3
		NM_152501	PYHIN1	NM_013338	ALG5
		NM_080701	TREX2	NM_024733	ZNF665
		NM_198443	NRN1L	NM_152573	RASEF
		NM_001080448	EPHA6	NM_005563	STMN1
		NM_020142	NDUFA4L2	NM_001201	BMP3
		NM_001168682	SAP25	NM_001247988	CTAGE5
		NM_001013732	PTCHD4	NM_005641	TAF6
		NM_001934	DLX4	NM_015246	MGRN1
10	**Heart (L. Ventricle)**	NM_001330161	ING5	NM_198475	FAM171A2
		NM_005952	MT1X	NM_001128844	SMARCA4
		NM_001285879	CEBPB	NM_030642	APOL5
		NM_014035	SNX24	NM_153711	FAM26E
		NM_001324201	EDA2R	NM_001300971	TBCB
		NM_145114	MAX	NM_016582	SLC15A3
		NM_001042747	FGR	NM_002275	KRT15
		NM_001301365	LYST	NM_002239	KCNJ3
		NM_001317063	WDR77	NM_173491	LSM11
		NM_015140	TTLL12	NM_005262	GFER
		NM_001127190	CSK	NM_000599	IGFBP5
		NM_001743	CALM2	NM_001190452	MTRNR2L1
		NM_198971	HINFP	NM_001206974	DHPS
		NM_003143	SSBP1	NM_003913	PRPF4B
		NM_144693	ZNF558	NM_016327	UPB1
		NM_006571	DCTN6	NM_001001922	OR52N5
		NM_020967	NCOA5	NM_024864	MRM1
		NM_018004	TMEM45A	NM_153362	PRSS35
		NM_001287509	PPCS	NM_001046	SLC12A2
		NM_001277115	DNAH11	NM_014548	TMOD2
		NM_001005498	RHBDF2	NM_052985	IFT122
		NM_001042410	ANKZF1	NM_182971	COX8C
		NM_006343	MERTK	NM_004079	CTSS
		NM_001002926	TWISTNB	NM_001172633	OLR1
		NM_173566	PRR14L	NM_001145658	RAP1GAP
		NM_001297617	ELOVL7	NM_001251991	GMNN

		NM_017577	GRAMD1C	NM_017559	FNDC8
		NM_001100880	ST20	NM_001308248	EVI5
		NM_198219	ING1	NM_003910	BUD31
		NM_182756	SPDYA	NM_001286655	DLK2
		NM_181701	QSOX2	NM_001284513	PAQR4
		NM_005872	BCAS2	NM_003273	TM7SF2
		NM_014175	MRPL15	NM_006638	RPP40
		NM_001243612	LMO3	NM_001330368	C11orf65
		NM_003841	TNFRSF10C	NM_020428	SLC44A2
		NM_001290403	TAL1	NM_178822	IGSF10
		NM_004954	MARK2	NM_001271888	CIB2
		NM_001099220	ZNF862	NM_001301687	GBX2
		NM_032687	CYHR1	NM_001136495	C1orf198
		NM_001324436	PARL	NM_001017920	DAPL1
		NM_015997	RRNAD1	NM_181656	C17orf58
		NM_005253	FOSL2	NM_020342	SLC39A10
		NM_006849	PDIA2	NM_000836	GRIN2D
		NM_014901	RNF44	NM_001287013	GLIPR2
		NM_001146019	TJAP1	NM_001174088	NCOA3
		NM_001042522	SPRED3	NM_014442	SIGLEC8
		NM_018837	SULF2	NM_006177	NRL
		NM_006606	RBBP9	NM_198426	CHMP2A
		NM_001012427	FOXP4	NM_001166018	POU6F2
		NM_001135021	ELMOD3	NM_024589	ROGDI
		NM_005015	OXA1L	NM_001297594	SEL1L3
		NM_006948	HSPA13	NM_023937	MRPL34
		NM_018218	USP40	NM_170674	MEIS2
		NM_213611	SLC25A3	NM_001010851	ZNF766
		NM_016052	RRP15	NM_000506	F2
		NM_002715	PPP2CA	NM_032403	PCDHGC3
		NM_130807	MOB3A	NM_003175	XCL2
		NM_018683	RNF114	NM_021636	LGR6
		NM_001039707	SDCCAG3	NM_153713	LIX1L
		NM_024830	LPCAT1	NM_000132	F8
		NM_015683	ARRDC2	NM_134441	RLN2
		NM_001292032	ZNF76	NM_005494	DNAJB6
		NM_021204	ENOPH1	NM_001272051	C16orf91
		NM_017612	ZCCHC8	NM_174909	TMEM167A
		NM_001309451	PLEKHB2	NM_024605	ARHGAP10
		NM_001130845	BCL6	NM_152391	PQLC3
		NM_001039709	CBFA2T2	NM_006398	UBD
		NM_001271808	TREML1	NM_001135037	MFAP3
		NM_001143980	CCDC154	NM_003507	FZD7
		NM_001185183	VAMP7	NM_015201	BOP1
		NM_001282112	TOP3B	NM_001282658	CCDC3
		NM_173627	ENDOV	NM_138972	BACE1
		NM_001330440	SMARCD2	NM_006842	SF3B2
		NM_002495	NDUFS4	NM_207359	GADL1
		NM_016354	SLCO4A1	NM_001184856	KLHDC4
		NM_001497	B4GALT1	NM_000815	GABRD
		NM_001146333	SUMF2	NM_001278924	ANXA8L1
		NM_001330246	METAP2	NM_001291462	TBC1D3G
		NM_001300963	CDK19	NM_032775	KLHL22
		NM_022767	AEN	NM_000253	MTTP

11	**Lung**	NM_001013732	PTCHD4	NM_006336	ZER1
		NM_001324201	EDA2R	NM_001193502	TCF24
		NM_022470	ZMAT3	NM_207045	ENSA
		NM_014214	IMPA2	NM_002909	REG1A
		NM_001271756	ITGBL1	NM_001184796	ESYT1
		NM_007021	C10orf10	NM_018402	IL26
		NM_052867	NALCN	NM_001167741	MTX3
		NM_001319174	FMO3	NM_205859	OR2K2
		NM_144584	HENMT1	NM_016817	OAS2
		NM_000492	CFTR	NM_022124	CDH23
		NM_203463	CERS6	NM_000778	CYP4A11
		NM_001005855	ATP8B2	NM_001083947	TMPRSS4
		NM_058195	CDKN2A	NM_001005327	OR6K3
		NM_025208	PDGFD	NM_003897	IER3
		NM_001142807	ACOXL	NM_001006607	LRRC37A2
		NM_015328	AHCYL2	NM_176818	GATC
		NM_024565	CCNJL	NM_001031723	DNAJB14
		NM_000758	CSF2	NM_001242672	TTC34
		NM_144612	LOXHD1	NM_152572	AK8
		NM_001032295	SERPING1	NM_002192	INHBA
		NM_001455	FOXO3	NM_019048	ASNSD1
		NM_001463	FRZB	NM_177552	SULT1A3
		NM_001201	BMP3	NM_001271441	C21orf2
		NM_001317858	CHCHD7	NM_001679	ATP1B3
		NM_005928	MFGE8	NM_001177433	TRPV4
		NM_001323369	WISP2	NM_001037500	DEFB124
		NM_052916	RNF157	NM_001154	ANXA5
		NM_001134451	TMEM130	NM_002995	XCL1
		NM_198526	ZNF710	NM_001286814	TNFAIP8
		NM_018218	USP40	NM_001134408	GRIN2A
		NM_001308395	EFHD1	NM_014465	SULT1B1
		NM_205834	LSR	NM_001080551	C9orf84
		NM_018936	PCDHB2	NM_014704	CEP104
		NM_001258312	PDE1A	NM_000943	PPIC
		NM_173808	NEGR1	NM_001242789	BRF1
		NM_021076	NEFH	NM_002153	HSD17B2
		NM_201442	C1S	NM_004118	FOXS1
		NM_001166110	PALLD	NM_018945	PDE7B
		NM_001166280	MUSK	NM_002356	MARCKS
		NM_005907	MAN1A1	NM_001032284	TMPO
		NM_019119	PCDHB9	NM_022075	CERS2
		NM_001291977	SPARCL1	NM_001289177	ACSBG2
		NM_002428	MMP15	NM_001304275	RAPGEF1
		NM_007231	SLC6A14	NM_001077621	VPS37D
		NM_001018057	DKK3	NM_002271	IPO5
		NM_014483	RBMS3	NM_138381	OXNAD1
		NM_021969	NR0B2	NM_172169	CAMK2G
		NM_001305204	ZFP90	NM_001243108	PLD2
		NM_054025	B3GAT1	NM_004421	DVL1
		NM_001322802	NNAT	NM_181291	WDR20
		NM_001129	AEBP1	NM_018346	RSAD1
		NM_002657	PLAGL2	NM_001098522	HTATIP2
		NM_145691	ATPAF2	NM_001080466	BTBD17
		NM_002944	ROS1	NM_015868	KIR2DL3
		NM_001144978	MTHFD2L	NM_024528	NKAP
		NM_001143981	CHRDL1	NM_057090	ARTN

		NM_153366	SVEP1	NM_012408	ZMYND8
		NM_017708	FAM83E	NM_001204077	UBE4A
		NM_018930	PCDHB10	NM_020399	GOPC
		NM_024010	MTRR	NM_001145636	C1orf228
		NM_019050	USP53	NM_020371	AVEN
		NM_001291454	ATP2C2	NM_001306089	ZNF236
		NM_001324101	EBF1	NM_001159560	BEX5
		NM_014426	SNX5	NM_173163	NFATC3
		NM_003630	PEX3	NM_021163	RBAK
		NM_144617	HSPB6	NM_003170	SUPT6H
		NM_001247996	ASAP1	NM_001164232	DDHD2
		NM_000297	PKD2	NM_014856	DENND4B
		NM_001330188	TBC1D30	NM_183050	BCKDHB
		NM_001012994	SNX30	NM_001353	AKR1C1
		NM_001172743	RAI2	NM_001080513	CPN2
		NM_024581	FAM184A	NM_181538	GJC3
		NM_016258	YTHDF2	NM_001253853	AP4B1
		NM_014398	LAMP3	NM_018986	SH3TC1
		NM_001304444	ALAS1	NM_012413	QPCT
		NM_020453	ATP10D	NM_146421	GSTM1
		NM_019027	RBM47	NM_174899	FBXO36
		NM_015205	ATP11A	NM_023009	MARCKSL1
		NM_017700	ARHGEF38	NM_207380	C15orf52
		NM_001261391	CALCOCO2	NM_144582	TEX261
12	**Muscle (Skeletal)**	NM_001080534	UNC13C	NM_001301103	PPCDC
		NM_001277115	DNAH11	NM_001277201	SIGLEC7
		NM_001304939	GPATCH8	NM_018425	PI4K2A
		NM_012307	EPB41L3	NM_006533	MIA
		NM_001193466	KANSL1	NM_014442	SIGLEC8
		NM_004110	FDXR	NM_001145045	ZNF286B
		NM_001303468	SKAP2	NM_006108	SPON1
		NM_001010924	FAM171A1	NM_003014	SFRP4
		NM_001320044	SCAP	NM_138705	CALML6
		NM_001136040	CPSF7	NM_001145785	MEF2B
		NM_001034845	GALNTL6	NM_173846	TPPP2
		NM_022095	ZNF335	NM_000196	HSD11B2
		NM_015574	ANKRD17	NM_004797	ADIPOQ
		NM_001297428	TMEM175	NM_004065	CDR1
		NM_014516	CNOT3	NM_199350	C9orf50
		NM_005599	NHLH2	NM_015694	ZNF777
		NM_001300886	RHOD	NM_002490	NDUFA6
		NM_145059	FUK	NM_001243386	PARVB
		NM_003743	NCOA1	NM_006342	TACC3
		NM_014733	ZFYVE16	NM_030589	CYP2A7
		NM_001005332	MAGED1	NM_001097622	OCM
		NM_181872	DMRT2	NM_152379	C1orf131
		NM_198514	NHLRC2	NM_001308147	PLEKHG3
		NM_000911	OPRD1	NM_014420	DKK4
		NM_001324201	EDA2R	NM_001318722	KBTBD4
		NM_002503	NFKBIB	NM_001277163	CEACAM3
		NM_017637	BNC2	NM_014688	USP6NL
		NM_020666	CLK4	NM_006802	SF3A3
		NM_182498	ZNF428	NM_024011	CDK11A
		NM_016333	SRRM2	NM_033315	RASL10B
		NM_001129908	FAM198A	NM_001293296	MYEOV
		NM_198184	OSTN	NM_001282962	HJURP

		NM_001330651	ARHGAP36	NM_018365	MNS1
		NM_181808	POLN	NM_001316327	PRKCD
		NM_012117	CBX5	NM_199133	FAM173B
		NM_001288989	PLCE1	NM_000436	OXCT1
		NM_003204	NFE2L1	NM_021818	SAV1
		NM_001013659	ZNF793	NM_006636	MTHFD2
		NM_005107	EXOG	NM_024683	TEFM
		NM_001079526	IKZF2	NM_001330230	IFI35
		NM_001127715	STXBP5	NM_002085	GPX4
		NM_203463	CERS6	NM_021920	SCT
		NM_022497	MRPS25	NM_000306	POU1F1
		NM_015267	CUX2	NM_015205	ATP11A
		NM_006231	POLE	NM_016353	ZDHHC2
		NM_001184782	CXorf57	NM_173806	PDZD9
		NM_001330694	CEP78	NM_015944	AMDHD2
		NM_016238	ANAPC7	NM_000189	HK2
		NM_001321316	MKLN1	NM_001629	ALOX5AP
		NM_020461	TUBGCP6	NM_022353	OSGEPL1
		NM_014225	PPP2R1A	NM_001184700	UGDH
		NM_001321229	HDAC6	NM_001261441	EXTL2
		NM_003064	SLPI	NM_006704	SUGT1
		NM_001013732	PTCHD4	NM_004494	HDGF
		NM_001321458	REV1	NM_001083965	TDRKH
		NM_020410	ATP13A1	NM_033225	CSMD1
		NM_004273	CHST3	NM_001105520	C17orf100
		NM_001242539	NCOA6	NM_001109977	FAM160A1
		NM_005500	SAE1	NM_004776	B4GALT5
		NM_001303457	TTI1	NM_032796	SYAP1
		NM_020964	EPG5	NM_001169574	CXorf58
		NM_174922	ADCK5	NM_000887	ITGAX
		NM_032289	PSD2	NM_016541	GNG13
		NM_001303141	PDZRN3	NM_006244	PPP2R5B
		NM_001160227	SPG11	NM_030802	FAM117A
		NM_001329434	NUP205	NM_001817	CEACAM4
		NM_001277313	FMN1	NM_001040146	CHTF8
		NM_001142966	GREB1L	NM_174983	MFSD12
		NM_017887	C1orf123	NM_000706	AVPR1A
		NM_014271	IL1RAPL1	NM_001288711	ZSCAN30
		NM_001244714	FGGY	NM_138733	PGK2
		NM_080413	VPS16	NM_145256	LRRC25
		NM_001858	COL19A1	NM_001143943	EFCAB2
		NM_031209	QTRT1	NM_033631	LUZP1
		NM_145263	SPATA18	NM_003491	NAA10
		NM_016376	ANKFY1	NM_001168298	CXCR2
		NM_001287759	COL4A6	NM_005627	SGK1
		NM_012272	PRPF40B	NM_001277307	MAGEB17
		NM_015995	KLF13	NM_001033569	AMZ2
		NM_178136	POLDIP3	NM_004387	NKX2-5
13	**Nerve (Tibia)**	NM_001324201	EDA2R	NM_145276	ZNF563
		NM_000332	ATXN1	NM_178134	CYP4Z1
		NM_182767	SLC6A15	NM_017639	DCHS2
		NM_001042520	C2orf88	NM_173076	ABCA12
		NM_021973	HAND2	NM_000303	PMM2
		NM_152501	PYHIN1	NM_001190447	PPP2R3A
		NM_022470	ZMAT3	NM_001198828	LIPF
		NM_001242813	ANKRD6	NM_030578	B9D2

	NM_015488	PNKD	NM_013318	PRRC2B
	NM_175062	RASGEF1C	NM_001170460	CDK16
	NM_058195	CDKN2A	NM_001042633	SNX21
	NM_001303103	TLE1	NM_182541	TMEM31
	NM_002889	RARRES2	NM_005245	FAT1
	NM_001291721	CYFIP2	NM_001318875	FLOT1
	NM_001199295	ZNF549	NM_016403	CWC15
	NM_001242763	DLGAP1	NM_001146267	GPR85
	NM_031474	NRIP2	NM_005801	EIF1
	NM_001287438	COBL	NM_001170765	LCOR
	NM_182511	CBLN2	NM_152516	COMMD1
	NM_002338	LSAMP	NM_001167865	NSMCE4A
	NM_001135940	MYOT	NM_000986	RPL24
	NM_016588	NRN1	NM_001306195	ARPP19
	NM_012301	MAGI2	NM_001324177	ZNF33A
	NM_003848	SUCLG2	NM_003907	EIF2B5
	NM_017921	NPLOC4	NM_130474	MADD
	NM_001253693	MCOLN3	NM_001135697	SGCA
	NM_152423	MUM1L1	NM_001170714	BCAR1
	NM_181351	NCAM1	NM_022836	DCLRE1B
	NM_018557	LRP1B	NM_018304	PRR11
	NM_002775	HTRA1	NM_001282776	POLR1B
	NM_001171184	DRP2	NM_005815	ZNF443
	NM_001282716	STAG3	NM_012215	MGEA5
	NM_022460	HS1BP3	NM_001317951	BRD9
	NM_012168	FBXO2	NM_001247996	ASAP1
	NM_014747	RIMS3	NM_002439	MSH3
	NM_001271213	SQRDL	NM_001099652	GPR137C
	NM_002213	ITGB5	NM_001271640	ZNF138
	NM_183404	RBL1	NM_002923	RGS2
	NM_003106	SOX2	NM_033413	LRRC46
	NM_001320634	BIN1	NM_021248	CDH22
	NM_004962	GDF10	NM_003451	ZNF177
	NM_016609	SLC22A17	NM_006820	IFI44L
	NM_199037	SCN1B	NM_014420	DKK4
	NM_001001331	ATP2B2	NM_001202457	ZNF816
	NM_002228	JUN	NM_001242867	KIR3DL2
	NM_000425	L1CAM	NM_018990	SASH3
	NM_021076	NEFH	NM_001126336	VCAN
	NM_005116	SLC23A2	NM_181715	CRTC2
	NM_004466	GPC5	NM_007163	SLC14A2
	NM_001122841	FRMD1	NM_032496	ARHGAP9
	NM_006558	KHDRBS3	NM_013436	NCKAP1
	NM_004490	GRB14	NM_001001936	AFAP1L2
	NM_001201461	TTYH1	NM_001172654	LILRA3
	NM_001265615	FAM153B	NM_152792	ASPRV1
	NM_001080480	MBOAT1	NM_003196	TCEA3
	NM_053043	RBM33	NM_001301189	LINGO1
	NM_033402	LRRCC1	NM_018229	AP5M1
	NM_001324014	CORO2B	NM_001244683	PDE3A
	NM_018190	BBS7	NM_015024	XPO7
	NM_004110	FDXR	NM_001142550	WDR47
	NM_002148	HOXD10	NM_001038640	GOLGA6A
	NM_001992	F2R	NM_001243597	CDON
	NM_001346083	CNTN2	NM_198279	CXorf23
	NM_001039703	NBPF10	NM_001321830	DDX41

		NM_004540	NCAM2	NM_012467	TPSG1
		NM_032995	ARHGEF4	NM_006821	ACOT2
		NM_001013732	PTCHD4	NM_013403	STRN4
		NM_001199399	NEK1	NM_001034996	RPL14
		NM_001077269	WIPF1	NM_018198	DNAJC11
		NM_006210	PEG3	NM_004087	DLG1
		NM_020820	PREX1	NM_005868	BET1
		NM_001206838	PTPRZ1	NM_005454	CER1
		NM_001167880	LHPP	NM_001195556	CLINT1
		NM_002015	FOXO1	NM_001272054	MRFAP1
		NM_001256550	MPP5	NM_012263	TTLL1
		NM_052902	STK11IP	NM_016417	GLRX5
		NM_001278562	LCTL	NM_138568	EXOC3L2
		NM_006248	PRB2	NM_001317980	RPL26L1
		NM_012443	SPAG6	NM_005708	GPC6
		NM_001281747	MLIP	NM_006355	TRIM38
14	**Skin I (Lower Leg)**	NM_153281	HYAL1	NM_001317029	ZNF418
		NM_017754	UHRF1BP1	NM_006963	ZNF22
		NM_001301007	AMOTL1	NM_001142389	PNPLA4
		NM_018936	PCDHB2	NM_000777	CYP3A5
		NM_014272	ADAMTS7	NM_018912	PCDHGA1
		NM_033632	FBXW7	NM_001014840	CUTA
		NM_001114634	PLAG1	NM_001321704	RPS9
		NM_014997	KLHDC10	NM_000953	PTGDR
		NM_001319174	FMO3	NM_145062	ZUFSP
		NM_001271856	GRASP	NM_018371	CSGALNACT1
		NM_015902	UBR5	NM_031296	RAB33B
		NM_033631	LUZP1	NM_139165	RAET1E
		NM_001013253	LSP1	NM_033484	FBXO4
		NM_007021	C10orf10	NM_020361	CPA6
		NM_015562	UBXN7	NM_016403	CWC15
		NM_052943	FAM46B	NM_018180	DHX32
		NM_152594	SPRED1	NM_001318502	INPP5E
		NM_001199295	ZNF549	NM_006794	GPR75
		NM_001136557	GPR107	NM_001008269	TMEM89
		NM_018384	GIMAP5	NM_002911	UPF1
		NM_001014279	ANXA2R	NM_020321	ASIC3
		NM_003966	SEMA5A	NM_002964	S100A8
		NM_152501	PYHIN1	NM_001318900	GLUD1
		NM_017986	SLC52A1	NM_003329	TXN
		NM_001171193	GDPD2	NM_183377	ASIC2
		NM_001170905	ZNF736	NM_001402	EEF1A1
		NM_001145078	ZNF805	NM_001133	AFM
		NM_145282	SLC25A48	NM_014324	AMACR
		NM_001258463	PAX6	NM_015073	SIPA1L3
		NM_001320559	CSRNP1	NM_000314	PTEN
		NM_173480	ZNF57	NM_006580	CLDN16
		NM_030641	APOL6	NM_001145934	TKTL1
		NM_004963	GUCY2C	NM_001288728	PPRC1
		NM_001243612	LMO3	NM_001330071	DCLK1
		NM_001099678	LRRC58	NM_001135195	SLC39A5
		NM_058238	WNT7B	NM_021238	FAM60A
		NM_001099270	ZBTB34	NM_003526	HIST1H2BC
		NM_000773	CYP2E1	NM_001135196	C10orf71
		NM_178559	ABCB5	NM_001145678	KIAA0825
		NM_020354	ENTPD7	NM_001318045	FOLR3

		NM_000428	LTBP2	NM_001006656	ZNF473
		NM_001286414	TUBGCP4	NM_017584	MIOX
		NM_138455	CTHRC1	NM_001170649	LAS1L
		NM_020377	CYSLTR2	NM_004345	CAMP
		NM_016270	KLF2	NM_000206	IL2RG
		NM_030761	WNT4	NM_139052	TAF5
		NM_001320621	GRIK1	NM_003954	MAP3K14
		NM_001034077	HIST2H4B	NM_001293204	MVP
		NM_001272013	ITPRIP	NM_175734	C17orf74
		NM_001427	EN2	NM_025224	ZBTB46
		NM_144775	SMCR8	NM_024685	BBS10
		NM_030753	WNT3	NM_002043	GABRR2
		NM_194314	ZBTB41	NM_024874	KIAA0319L
		NM_001144890	SLC23A3	NM_001272069	ABHD10
		NM_022454	SOX17	NM_014049	ACAD9
		NM_183416	KIF1B	NM_182644	EPHA3
		NM_015714	G0S2	NM_144769	FOXI1
		NM_001080392	KIAA1147	NM_138690	GRIN3B
		NM_001166412	SMOC2	NM_020665	TMEM27
		NM_025106	SPSB1	NM_020762	SRGAP1
		NM_001282458	C2	NM_001185113	CD1E
		NM_024963	FBXL18	NM_006265	RAD21
		NM_006403	NEDD9	NM_001004358	FGFRL1
		NM_001297734	ZNF417	NM_000701	ATP1A1
		NM_178554	KY	NM_001001524	TM6SF2
		NM_001193508	REST	NM_144628	TBC1D20
		NM_130440	PTPRF	NM_003816	ADAM9
		NM_001261830	SERPINB7	NM_014575	SCHIP1
		NM_001281492	MSH6	NM_181333	PRR5
		NM_052958	C8orf34	NM_006114	TOMM40
		NM_016248	AKAP11	NM_006914	RORB
		NM_001316968	C5orf30	NM_175873	SOWAHA
		NM_004397	DDX6	NM_173582	PGM2L1
		NM_001032391	LCMT1	NM_013386	SLC25A24
		NM_016270	KLF2	NM_001318362	EXOSC1
15	**Skin II-a (M, Upper Arm)**	NM_000240	MAOA	NM_000088	COL1A1
		NM_000287	PEX6	NM_000275	OCA2
		NM_000487	ARSA	NM_000304	PMP22
		NM_000543	SMPD1	NM_000372	TYR
		NM_000781	CYP11A1	NM_000373	UMPS
		NM_000837	GRINA	NM_000530	MPZ
		NM_001033551	TOM1L2	NM_001001994	GPM6B
		NM_001089	ABCA3	NM_001007023	DIO2
		NM_002735	PRKAR1B	NM_001007538	SHISA2
		NM_003224	ARFRP1	NM_001008223	C1QL4
		NM_003560	PLA2G6	NM_001010898	SLC6A17
		NM_003586	DOC2A	NM_001014447	CPZ
		NM_003708	RDH16	NM_001029858	SLC35F1
		NM_003748	ALDH4A1	NM_001167	XIAP
		NM_003782	B3GALT4	NM_001248	ENTPD3
		NM_003801	GPAA1	NM_001609	ACADSB
		NM_004224	GPR50	NM_002072	GNAQ
		NM_004364	CEBPA	NM_002594	PCSK2
		NM_004606	TAF1	NM_002600	PDE4B
		NM_005488	TOM1	NM_002677	PMP2
		NM_006026	H1FX	NM_003507	FZD7

	NM_006152	LRMP	NM_003618	MAP4K3
	NM_006383	CIB2	NM_003833	MATN4
	NM_006648	WNK2	NM_004071	CLK1
	NM_006869	ADAP1	NM_004378	CRABP1
	NM_007079	PTP4A3	NM_004472	FOXD1
	NM_012190	ALDH1L1	NM_004789	LHX2
	NM_013992	PAX8	NM_006087	TUBB4A
	NM_014690	FAM131B	NM_006198	PCP4
	NM_014826	CDC42BPA	NM_006364	SEC23A
	NM_015321	CRTC1	NM_006587	CORIN
	NM_015516	TSKU	NM_006890	CEACAM7
	NM_015944	AMDHD2	NM_007191	WIF1
	NM_016002	SCCPDH	NM_007281	SCRG1
	NM_016539	SIRT6	NM_012124	CHORDC1
	NM_017777	MKS1	NM_012425	RSU1
	NM_017931	TTC38	NM_012469	PRPF6
	NM_018378	FBXL8	NM_013377	PDZRN4
	NM_018402	IL26	NM_014789	ZNF623
	NM_019058	DDIT4	NM_014862	ARNT2
	NM_020439	CAMK1G	NM_014951	ZNF365
	NM_022307	ICA1	NM_015097	CLASP2
	NM_023942	LRRC61	NM_015358	MORC3
	NM_024042	METRN	NM_015464	SOSTDC1
	NM_024043	DBNDD1	NM_016180	SLC45A2
	NM_024302	MMP28	NM_016216	DBR1
	NM_024722	ACBD4	NM_016374	ARID4B
	NM_030662	MAP2K2	NM_018375	SLC39A9
	NM_032017	STK40	NM_019845	RPRM
	NM_032219	MFSD7	NM_020747	ZNF608
	NM_032272	MAF1	NM_021948	BCAN
	NM_032370	ZNF414	NM_022068	PIEZO2
	NM_032630	CINP	NM_022122	MMP27
	NM_032789	PARP10	NM_022469	GREM2
	NM_080621	SAMD10	NM_022841	RFX7
	NM_080749	NEURL2	NM_022843	PCDH20
	NM_133467	CITED4	NM_023940	RASL11B
	NM_138355	SCRN2	NM_024803	TUBAL3
	NM_138570	SLC38A10	NM_025074	FRAS1
	NM_138612	HAS3	NM_030590	MATN4
	NM_138778	DPH7	NM_030660	ATXN3
	NM_144668	WDR66	NM_031866	FZD8
	NM_145071	CISH	NM_032246	MEX3B
	NM_152236	GAS2L1	NM_033102	SLC45A3
	NM_152246	CPT1B	NM_033119	NKD1
	NM_152353	CLDND2	NM_139160	DEPDC7
	NM_152544	TRMT44	NM_145312	ZNF485
	NM_152677	ZSCAN4	NM_152730	TBC1D32
	NM_152718	VWCE	NM_152780	MAP7D2
	NM_152911	PAOX	NM_153321	PMP22
	NM_170726	ALDH4A1	NM_153464	ILF3
	NM_175078	KRT77	NM_153809	TAF1L
	NM_178232	HAPLN3	NM_173462	PAPLN
	NM_182530	MYRFL	NM_173607	FAM177A1
	NM_183373	PXDC1	NM_178233	OTOP3
	NM_198533	HSD11B1L	NM_181783	TMTC3
	NM_206920	MAMDC4	NM_198887	NUP43

		NM_206967	C16orf74	NM_198990	NAPEPLD
16	**Skin II-b (F, Upper Arm)**	NM_000156	GAMT	NM_000088	COL1A1
		NM_000419	ITGA2B	NM_000089	COL1A2
		NM_000804	FOLR3	NM_000090	COL3A1
		NM_001011649	CDK5RAP2	NM_000130	F5
		NM_001017392	SUGP2	NM_000393	COL5A2
		NM_001018	RPS15	NM_000396	CTSK
		NM_001033551	TOM1L2	NM_001001991	PAMR1
		NM_001654	ARAF	NM_001003942	BMF
		NM_002477	MYL5	NM_001007538	SHISA2
		NM_002828	PTPN2	NM_001014447	CPZ
		NM_003314	TTC1	NM_001452	FOXF2
		NM_003549	HYAL3	NM_001458	FLNC
		NM_003708	RDH16	NM_001797	CDH11
		NM_003752	EIF3C	NM_001848	COL6A1
		NM_004019	DMD	NM_001935	DPP4
		NM_004435	ENDOG	NM_002148	HOXD10
		NM_004564	GATB	NM_002223	ITPR2
		NM_004707	ATG12	NM_002514	NOV
		NM_004722	AP4M1	NM_002593	PCOLCE
		NM_005123	NR1H4	NM_003013	SFRP2
		NM_005309	GPT	NM_003062	SLIT3
		NM_005331	HBQ1	NM_003250	THRA
		NM_005630	SLCO2A1	NM_003392	WNT5A
		NM_005687	FARSB	NM_003505	FZD1
		NM_006293	TYRO3	NM_003507	FZD7
		NM_006702	PNPLA6	NM_003833	MATN4
		NM_006987	RPH3AL	NM_003881	WISP2
		NM_007254	PNKP	NM_004098	EMX2
		NM_014702	KIAA0408	NM_004385	VCAN
		NM_015071	ARHGAP26	NM_004460	FAP
		NM_016173	HEMK1	NM_004530	MMP2
		NM_016539	SIRT6	NM_004554	NFATC4
		NM_017539	DNAH3	NM_004655	AXIN2
		NM_017570	OPLAH	NM_005012	ROR1
		NM_017914	C19orf24	NM_005014	OMD
		NM_017984	ZCWPW1	NM_005290	GPR15
		NM_019624	ABCB9	NM_005406	ROCK1
		NM_020299	AKR1B10	NM_005602	CLDN11
		NM_020386	HRASLS	NM_006039	MRC2
		NM_020421	ADCK1	NM_006206	PDGFRA
		NM_021021	SNTB1	NM_006587	CORIN
		NM_021075	NDUFV3	NM_006682	FGL2
		NM_022350	ERAP2	NM_007191	WIF1
		NM_024043	DBNDD1	NM_007270	FKBP9
		NM_024100	WDR18	NM_014817	TRIL
		NM_024682	TBC1D17	NM_014914	AGAP1
		NM_024803	TUBAL3	NM_015696	GPX7
		NM_024944	CHODL	NM_016174	CERCAM
		NM_025149	ACSF2	NM_017680	ASPN
		NM_032687	CYHR1	NM_020742	NLGN4X
		NM_032788	ZNF514	NM_020774	MIB1
		NM_052845	MMAB	NM_021110	COL14A1
		NM_052957	GCNA	NM_021785	RAI2
		NM_133436	ASNS	NM_021983	HLA-DRB4
		NM_138384	MTG1	NM_022122	MMP27

		NM_138431	MFSD3	NM_024697	ZNF385D
		NM_138465	GLI4	NM_030590	MATN4
		NM_139266	STAT1	NM_032777	ADGRA2
		NM_144666	DNHD1	NM_033274	ADAM19
		NM_145170	CFAP70	NM_052913	TMEM200A
		NM_145276	ZNF563	NM_130830	LRRC15
		NM_152246	CPT1B	NM_130848	DCANP1
		NM_152474	C19orf18	NM_133459	CCBE1
		NM_172312	SPAG8	NM_138440	VASN
		NM_176792	MRPL43	NM_138636	TLR8
		NM_178120	DLX1	NM_139125	MASP1
		NM_178545	TMEM52	NM_145165	CHURC1
		NM_181843	NUDT8	NM_145305	SLC25A43
		NM_198271	LMOD3	NM_153256	PROSER2
		NM_198562	C3orf62	NM_153267	MAMDC2
		NM_207340	ZDHHC24	NM_153370	PI16
		NM_207351	PRRT3	NM_173490	TMEM171
		NM_001012665	DYNC2LI1	NM_173833	SCARA5
		NM_001013701	DUXAP10	NM_181435	C1QTNF3
		NM_001017389	SULT1A4	NM_183377	ASIC2
				NM_199512	CCDC80
17	**Thyroid**	NM_001013732	PTCHD4	NM_198526	ZNF710
		NM_001173467	SP7	NM_021067	GINS1
		NM_022470	ZMAT3	NM_014793	LCMT2
		NM_002318	LOXL2	NM_001320700	ANXA4
		NM_001406	EFNB3	NM_001184743	PGBD1
		NM_022897	RANBP17	NM_001131026	PEX5
		NM_152419	HGSNAT	NM_003167	SULT2A1
		NM_004982	KCNJ8	NM_138288	SPTSSA
		NM_001315535	PDE1B	NM_002150	HPD
		NM_001042698	ZSWIM7	NM_022131	CLSTN2
		NM_001320912	FXYD5	NM_003080	SMPD2
		NM_004864	GDF15	NM_001201	BMP3
		NM_000389	CDKN1A	NM_001243759	USP2
		NM_002202	ISL1	NM_002395	ME1
		NM_004093	EFNB2	NM_145648	SLC15A4
		NM_178460	SIRPD	NM_183239	GSTO2
		NM_016530	RAB8B	NM_006300	ZNF230
		NM_001256792	BMPR1B	NM_001164731	REEP1
		NM_018369	DEPDC1B	NM_001306093	ZADH2
		NM_001243774	GNG2	NM_001317851	HEMK1
		NM_000239	LYZ	NM_001307924	NAP1L1
		NM_001199573	TRIM22	NM_000895	LTA4H
		NM_017743	DPP8	NM_001110781	SLC35E2B
		NM_005304	FFAR3	NM_001277066	MFF
		NM_001244071	GIMAP6	NM_006080	SEMA3A
		NM_001321103	SLC4A7	NM_016823	CRK
		NM_015941	ATP6V1H	NM_024545	SAP130
		NM_175060	CLEC14A	NM_005732	RAD50
		NM_001306076	CCDC18	NM_015186	VPS13A
		NM_001080383	GJC1	NM_182935	MOBP
		NM_145263	SPATA18	NM_003656	CAMK1
		NM_001113397	ZNF385B	NM_001142806	SLC6A8
		NM_014909	VASH1	NM_001169	AQP8
		NM_001199637	ADCYAP1R1	NM_007187	WBP4
		NM_001302998	LIPI	NM_003647	DGKE

		NM_001301012	EYA4	NM_001098626	ZNF98
		NM_006516	SLC2A1	NM_001966	EHHADH
		NM_053042	ZNF518B	NM_001017989	OPA3
		NM_001301129	POLR2F	NM_020223	FAM20C
		NM_153236	GIMAP7	NM_000660	TGFB1
		NM_006914	RORB	NM_005446	P2RX6
		NM_138435	FAM83F	NM_001304444	ALAS1
		NM_013241	FHOD1	NM_002751	MAPK11
		NM_014517	UBP1	NM_001114632	JMJD7
		NM_016002	SCCPDH	NM_001279350	REXO4
		NM_001330453	TTYH2	NM_003704	FAM193A
		NM_019858	GPR162	NM_152850	PIGO
		NM_002001	FCER1A	NM_152445	FAM161B
		NM_018420	SLC22A15	NM_017905	TMCO3
		NM_057168	WNT16	NM_177454	FAM171B
		NM_001300814	KRT78	NM_000327	ROM1
		NM_001291721	CYFIP2	NM_001173487	NKRF
		NM_001330238	MYO1B	NM_005325	HIST1H1A
		NM_006602	TCFL5	NM_016255	FAM8A1
		NM_001080457	LRRC4B	NM_025249	KIAA1683
		NM_004588	SCN2B	NM_017933	PID1
		NM_001297760	ETNK2	NM_001144037	TMEM25
		NM_015871	ZNF593	NM_001291428	BAX
		NM_001099439	EPHA10	NM_012329	MMD
		NM_005018	PDCD1	NM_001012975	RNASE10
		NM_002609	PDGFRB	NM_001308248	EVI5
		NM_013451	MYOF	NM_001006610	SIAH1
		NM_012168	FBXO2	NM_001319182	RNPEP
		NM_001323343	AHCTF1	NM_001282587	H6PD
		NM_001145399	MPPED2	NM_001012302	ANO9
		NM_025194	ITPKC	NM_198699	KRTAP10-12
		NM_201552	FGL1	NM_006708	GLO1
		NM_032305	POLR3GL	NM_153266	TMEM151A
		NM_001184960	SH3KBP1	NM_024082	PRRG3
		NM_000879	IL5	NM_002989	CCL21
		NM_173500	TTBK2	NM_001134938	FIP1L1
		NM_005763	AASS	NM_001164673	DOK7
		NM_139265	EHD4	NM_173791	PDZD8
		NM_001080470	ZNF697	NM_001024383	NAV3
		NM_001135031	GFI1B	NM_152329	LRR1
		NM_031310	PLVAP	NM_001136483	C17orf105
		NM_001255976	PMEPA1	NM_001374	DNASE1L2
		NM_001286692	RGS22	NM_145858	CRYZL1
		NM_006931	SLC2A3	NM_001032396	PJA1
		NM_016125	RNFT1	NM_012247	SEPHS1
18	**Blood**	NM_001144945	MYL12B	NM_001102614	SLC35G6
		NM_001184795	PEX11B	NM_000769	CYP2C19
		NM_001123369	PPP6C	NM_000086	CLN3
		NM_001084	PLOD3	NM_033517	SHANK3
		NM_018440	PAG1	NM_001424	EMP2
		NM_002811	PSMD7	NM_015419	MXRA5
		NM_144999	LRRC45	NM_014402	UQCRQ
		NM_006828	ASCC3	NM_176818	GATC
		NM_001160223	RNF170	NM_012342	BAMBI
		NM_030792	GDPD5	NM_001282447	ADAM33
		NM_003130	SRI	NM_022058	SLC4A10

		NM_058195	CDKN2A	NM_006662	SRCAP
		NM_018423	STYK1	NM_032827	ATOH8
		NM_001324074	CDK7	NM_001284513	PAQR4
		NM_033121	ANKRD13A	NM_033111	N4BP2L2
		NM_001303513	PDZD4	NM_024411	PDYN
		NM_178831	GATS	NM_002026	FN1
		NM_002802	PSMC1	NM_182826	SCARA3
		NM_001080412	ZBTB38	NM_001254717	DAXX
		NM_181575	AUP1	NM_153216	POU5F2
		NM_015622	CCZ1	NM_001252100	KIF21B
		NM_001308080	COMMD10	NM_001329998	TRANK1
		NM_152321	ERP27	NM_001135212	FKBP7
		NM_001135914	KCP	NM_001080470	ZNF697
		NM_001294282	MYBL1	NM_020992	PDLIM1
		NM_006671	SLC1A7	NM_001255985	COLEC11
		NM_001287342	PHPT1	NM_015041	CLUAP1
		NM_001142864	PIEZO1	NM_001272051	C16orf91
		NM_001081955	RGS9	NM_005236	ERCC4
		NM_001277066	MFF	NM_001282201	ZNF630
		NM_001278556	ARPC3	NM_003948	CDKL2
		NM_001173465	KIF21A	NM_001080434	LMTK3
		NM_007207	DUSP10	NM_139209	GRK7
		NM_001270399	TUBA1A	NM_001287212	KIF17
		NM_181719	TMCO4	NM_001164664	MAST4
		NM_145638	OSBPL5	NM_032437	EFCAB7
		NM_001308325	AKTIP	NM_001100603	KDELR2
		NM_003525	HIST1H2BI	NM_001297436	HAS1
		NM_016523	KLRF1	NM_004119	FLT3
		NM_001077665	AGAP6	NM_002043	GABRR2
		NM_020696	KIAA1143	NM_001277962	STIM1
		NM_001300771	LYPLAL1	NM_153613	LPCAT4
		NM_001170700	DTHD1	NM_005071	SLC1A6
		NM_001284217	FCRL6	NM_017443	POLE3
		NM_001071775	MZT1	NM_178171	GSDMA
		NM_001301716	CCR7	NM_207312	TUBA3E
		NM_001206836	RAB11A	NM_021080	DAB1
		NM_001286372	VTA1	NM_003281	TNNI1
		NM_001031803	LLGL2	NM_022049	GPR88
		NM_005347	HSPA5	NM_002987	CCL17
		NM_030768	ILKAP	NM_007028	TRIM31
		NM_001335	CTSW	NM_032139	ANKRD27
		NM_001039802	CDC42	NM_005996	TBX3
		NM_001142300	CCNYL1	NM_001183	ATP6AP1
		NM_003298	NR2C2	NM_003204	NFE2L1
		NM_021636	LGR6	NM_016932	SIX2
		NM_006575	MAP4K5	NM_001320151	OAS1
		NM_001252037	RAB5B	NM_032107	L3MBTL1
		NM_001305878	KAT6A	NM_003012	SFRP1
		NM_006621	AHCYL1	NM_001253823	BLVRA
		NM_001098801	FAM210A	NM_001318788	COX4I1
		NM_020840	FNIP2	NM_001164379	FAM180B
		NM_021928	SPCS3	NM_004164	RBP2
		NM_020133	AGPAT4	NM_000160	GCGR
		NM_001013836	MAD1L1	NM_172312	SPAG8
		NM_013379	DPP7	NM_001256398	BCO2
		NM_182523	CMC1	NM_018349	MCTP2

		NM_021204	ENOPH1	NM_001318027	PPP4R2
		NM_020474	GALNT1	NM_007127	VIL1
		NM_003958	RNF8	NM_001097611	KNCN
		NM_001162407	CLK1	NM_004984	KIF5A
		NM_001166345	MDFIC	NM_022450	RHBDF1
		NM_054025	B3GAT1	NM_080284	ABCA6
		NM_014905	GLS	NM_003883	HDAC3
		NM_001080414	CCDC88C	NM_013382	POMT2
		NM_001043353	TPM3	NM_182587	UNC80
		NM_024555	FBXL6	NM_000490	AVP
		NM_001195200	CCDC107	NM_022110	FKBPL
		NM_174922	ADCK5	NM_018176	LGI2
		NM_006627	POP4	NM_022475	HHIP
19	**Brain (Frontal Cortex)**	NM_000165	GJA1	NM_000724	CACNB2
		NM_000517	HBA2	NM_000801	FKBP1A
		NM_000518	HBB	NM_000814	GABRB3
		NM_000633	BCL2	NM_001001937	ATP5A1
		NM_000696	ALDH9A1	NM_001033581	PRKCZ
		NM_001018066	NTRK2	NM_001048	SST
		NM_001018161	PON2	NM_001113381	RGS4
		NM_001024	RPS21	NM_001114183	GRIA1
		NM_001039349	EFEMP1	NM_001131005	MEF2C
		NM_001126336	VCAN	NM_001134407	GRIN2A
		NM_001130082	PLXNB1	NM_001136262	ATXN7L3B
		NM_001163213	FGFR3	NM_001145648	RASGRF1
		NM_001166110	PALLD	NM_001165899	PDE4D
		NM_001387	DPYSL3	NM_001166392	TPBG
		NM_001400	S1PR1	NM_001171991	HCCS
		NM_001482	GATM	NM_001683	ATP2B2
		NM_001647	APOD	NM_001686	ATP5B
		NM_001954	DDR1	NM_002072	GNAQ
		NM_002193	INHBB	NM_002220	ITPKA
		NM_002221	ITPKB	NM_002576	PAK1
		NM_002303	LEPR	NM_002738	PRKCB
		NM_002645	PIK3C2A	NM_002739	PRKCG
		NM_002775	HTRA1	NM_002866	RAB3A
		NM_002998	SDC2	NM_003010	MAP2K4
		NM_003272	GPR137B	NM_003070	SMARCA2
		NM_003380	VIM	NM_003178	SYN2
		NM_003986	BBOX1	NM_003406	YWHAZ
		NM_004126	GNG11	NM_003885	CDK5R1
		NM_004781	VAMP3	NM_003969	UBE2M
		NM_004905	PRDX6	NM_004027	INPP4A
		NM_006005	WFS1	NM_004090	DUSP3
		NM_006108	SPON1	NM_004436	ENSA
		NM_006164	NFE2L2	NM_004588	SCN2B
		NM_006272	S100B	NM_004929	CALB1
		NM_006457	PDLIM5	NM_004958	MTOR
		NM_006621	AHCYL1	NM_005184	CALM3
		NM_012334	MYO10	NM_005249	FOXG1
		NM_013402	FADS1	NM_005389	PCMT1
		NM_014033	METTL7A	NM_005759	ABI2
		NM_014737	RASSF2	NM_005796	NUTF2
		NM_014899	RHOBTB3	NM_005909	MAP1B
		NM_014943	ZHX2	NM_006288	THY1
		NM_015166	MLC1	NM_006335	TIMM17A

		NM_015472	WWTR1	NM_006366	CAP2
		NM_015541	LRIG1	NM_012399	PITPNB
		NM_016235	GPRC5B	NM_013259	TAGLN3
		NM_018698	NXT2	NM_014231	VAMP1
		NM_018841	GNG12	NM_014452	TNFRSF21
		NM_018993	RIN2	NM_020309	SLC17A7
		NM_022359	PDE4DIP	NM_020977	ANK2
		NM_024060	AHNAK	NM_021032	FGF12
		NM_030935	TSC22D4	NM_021033	RAP2A
		NM_031442	TMEM47	NM_022893	BCL11A
		NM_033137	FGF1	NM_024294	C6orf106
		NM_145290	ADGRA3	NM_030671	PTPRO
		NM_152869	RGN	NM_031847	MAP2
		NM_153048	FYN	NM_032144	RAB6C
		NM_153186	KANK1	NM_033363	MRPS12
		NM_153367	ZCCHC24	NM_033642	FGF13
		NM_175617	MT1E	NM_057161	KLHDC3
		NM_177989	ACTL6A	NM_138282	ATP6V1G2
		NM_182943	PLOD2	NM_153341	RNF19B
		NM_198194	STOM	NM_176875	CCKBR
		NM_198951	TGM2	NM_181673	OGT
		NM_206855	QKI	NM_183416	KIF1B
		NM_213569	NEBL	NM_205768	ZBTB18
		NM_000071	CBS	NM_007030	TPPP
		NM_000186	CFH	NM_004958	MTOR
		NM_000533	PLP1	NM_004090	DUSP3
		NM_000540	RYR1	NM_012268	PLD3
		NM_001526	HCRTR2	NM_005610	RBB4
		NM_002210	ITGAV	NM_003338	UBE2D1
		NM_003748	ALDH4A1	NM_006095	ATP8A1
		NM_005397	PODXL	NM_004411	DYNC1I1
		NM_005806	OLIG2	NM_016841	MAPT
20	**Brain (Hippocampus)**	NM_152851	MS4A6A	NM_001467	SLC37A4
		NM_022349	MS4A6A	NM_001447	FAT2
		NM_001336	CTSZ	NM_021959	PPP1R11
		NM_001425	EMP3	NM_019848	SLC10A3
		NM_030818	CCDC130	NM_000175	GPI
		NM_052897	MBD6	NM_021045	ZNF248
		NM_001005619	ITGB4	NM_016020	TFB1M
		NM_174941	M160	NM_021928	SPCS3
		NM_001040021	CD14	NM_003645	SLC27A2
		NM_206938	MS4A7	NM_024544	C1orf166
		NM_013328	PYCR2	NM_004419	DUSP5
		NM_006790	MYOT	NM_015239	AGTPBP1
		NM_024610	HSPBAP1	NM_025225	PNPLA3
		NM_130841	ATP6V0A4	NM_021151	CROT
		NM_020461	TUBGCP6	NM_178863	KCTD13
		NM_003355	UCP2	NM_001042462	TRAPPC5
		NM_004121	GGT5	NM_003217	TMBIM6
		NM_005615	RNASE6	NM_022044	SDF2L1
		NM_007256	SLCO2B1	NM_052863	SCGB3A1
		NM_015409	EP400	NM_003953	MPZL1
		NM_000107	DDB2	NM_144575	CAPN13
		NM_001039706	C7orf63	NM_173854	SLC41A1
		NM_001457	FLNB	NM_025247	ACAD10
		NM_006371	CRTAP	NM_182492	LRP5L

		NM_016815	GYPC	NM_144573	NEXN
		NM_016234	ACSL5	NM_006110	CD2BP2
		NM_144601	CMTM3	NM_198687	KRTAP10-4
		NM_053042	ZNF518B	NM_003727	DNAH17
		NM_013372	GREM1	NM_001078645	CDC16
		NM_018460	ARHGAP15	NM_003124	SPR
		NM_004403	DFNA5	NM_000233	LHCGR
		NM_001001998	EXOSC10	NM_001002876	CENPM
		NM_032014	MRPS24	NM_018301	RBM41
		NM_031301	APH1B	NM_006043	HS3ST2
		NM_006312	NCOR2	NM_015869	PPARG
		NM_006682	FGL2	NM_020132	AGPAT3
		NM_001032409	OAS1	NM_022494	ZDHHC6
		NM_012432	SETDB1	NM_005797	MPZL2
		NM_012259	HEY2	NM_014647	KIAA0430
		NM_002944	ROS1	NM_014908	DOLK
		NM_003670	BHLHB2	NM_145331	MAP3K7
		NM_003332	TYROBP	NM_138411	FAM71E1
		NM_024096	DCTPP1	NM_001024649	CANX
		NM_005243	EWSR1	NM_080544	COLQ
		NM_000123	ERCC5	NM_000485	APRT
		NM_000621	HTR2A	XM_926671	KRTAP9-5
		NM_033554	HLA-DPA1	NM_152414	BHLHE22
		NM_003321	TUFM	NM_032227	TMEM164
		NM_001552	IGFBP4	NM_000943	PPIC
		NM_002340	LSS	NM_138787	C11orf74
		NM_016616	TXNDC3	NM_001083946	C2orf56
		NM_017907	C11orf59	NM_024076	KCTD15
		NM_001080526	FABP9	NM_181657	LTB4R
		NM_182495	FAM55B	NM_013246	CLCF1
		NM_003247	THBS2	NM_031455	CCDC3
		NM_138493	C6orf129	NM_005544	IRS1
		NM_000784	CYP27A1	NM_014384	ACAD8
		NM_005505	SCARB1	NM_001344	DAD1
		NM_003089	SNRNP70	NM_018660	ZNF395
		NM_022003	FXYD6	NM_006288	THY1
		NM_000599	IGFBP5	NM_005618	DLL1
		NM_018092	NETO2	NM_080632	UPF3B
		NM_003251	THRSP	NM_015510	DHRS7B
		NM_139353	TAF1C	NM_152644	FAM24B
		NM_001099786	ICAM2	NM_033557	YIF1B
		NM_030769	NPL	NM_001039673	YIF1B
		NM_016180	SLC45A2	NM_001031702	SEMA5B
		NM_015037	KIAA0913	NM_007008	RTN4
		NM_005950	MT1G	NM_002395	ME1
		NM_000920	PC	NM_014573	TMEM97
		NM_018664	BATF3	NM_003837	FBP2
		NM_133337	FER1L3	NM_004711	SYNGR1
		NM_001039651	C6orf26	NM_184085	TRIM55
		NM_138363	CCDC45	NM_007310	COMT
		NM_014623	MEA1	NM_198594	C1QTNF1
21	**Kidney**	NM_002127	HLA-G	NM_174938	MGC20553
		NM_006762	LAPTM5	NM_181788	H1FNT
		NM_004039	ANXA2	NM_153828	RTN4
		NM_002117	HLA-B	NM_001144969	NEDD4L
		NM_181481	C18orf1	NM_019044	FLJ10996

	NM_006169	NNMT	NM_015204	THSD7A
	NM_001025160	CD97	NM_017826	FLJ20449
	NM_004121	GGTLA1	NM_025057	BBOF1
	NM_015920	RPS27L	NM_000922	PDE3B
	NM_015892	GALNAC4S-6ST	NM_001171941	FRCP2
	NM_002546	TNFRSF11B	NM_001126044	PTGER3
	NM_002123	HLA-DQB1	NM_006044	HDAC6
	NM_015150	KIAA0084	NM_001003791	C8orf2
	NM_005516	HLA-E	NM_015984	UCHL5
	NM_001114101	C1QG	NM_138435	LOC113828
	NM_021983	HLA-DRB3	NM_001042463	TMEM80
	NM_001295	CCR1	NM_080927	ESDN
	NM_018950	HLA-F	NM_001719	BMP7
	NM_001166538	LST1	NM_021998	ZNF6
	NM_017554	KIAA1268	NM_213569	NEBL
	NM_020914	KIAA1554	NM_020340	KIAA1244
	NM_020954	KIAA1554	NM_152542	DKFZp761G058
	NM_199335	FYB	NM_021947	SRR
	NM_000591	CD14	NM_001017523	FLJ33957
	NM_022153	PP2135	NM_001584	C11orf8
	NM_003332	TYROBP	NM_199328	CLDN8
	NM_002117	HLA-C	NM_012175	FBXO3
	NM_033554	HLA-DPA1	NM_015602	MGC3413
	NM_000014	A2M	NM_000436	OXCT
	NM_001733	C1R	NM_001642	APLP2
	NM_012292	HA-1	NM_172232	ABCA5
	NM_199342	ERMAP	NM_207299	FLJ20300
	NM_052947	HAK	NM_001102416	KNG1
	NM_001166538	LST1	NM_001642	APLP2
	NM_018950	HLA-F	NM_031412	GABARAPL3
	NM_002649	PIK3CG	NM_001079529	KIAA0752
	NM_002127	HLA-G	NM_017512	HSRTSBETA
	NM_000491	C1QB	NM_006214	PHYH
	NM_021983	HLA-DRB1	NM_002896	RBM14
	NM_033554	HLA-DPA1	NM_001102416	KNG
	NM_002127	HLA-G	NM_152740	HIBADH
	NM_000586	IL-2	NM_152312	FLJ35207
	NM_015991	C1QA	NM_001625	FLJ10101
	NM_001134870	KIAA1949	NM_199040	NUDT4
	NM_001007469	PIK3AP	NM_000267	NF1
	NM_002117	HLA-C	NM_021047	ZNF253
	NM_012411	PTPN22	NM_015482	FLJ22174
	NM_001166538	LST1	NM_153828	RTN4
	NM_002123	HLA-DQB1	NM_001056	SULT1C1
	NM_002644	CDO1	NM_014510	PCLO
	NM_001167934	MGC4607	NM_015895	GMNN
	NM_012219	MRAS	NM_002567	PBP
	NM_002117	HLA-B	NM_001866	COX7B
	NM_207584	IFNAR2	NM_014007	ZNF297B
	NM_002117	HLA-B	NM_012343	NNT
	NM_003641	PTS	NM_001131018	CIZ1
	NM_201442	C1S	NM_016231	NLK
	NM_005211	CSF1R	NM_145804	DKFZP586C1619
	NM_019107	IL27w	NM_153696	PSMAL/GCP III
	NM_000211	ITGB2	NM_002300	LDHB
	NM_002423	MMP7	NM_001037540	SCML1

		NM_001166538	LST1	NM_000342	SLC4A1
		NM_001337	CX3CR1	NM_152542	DKFZp761G058
		NM_002127	HLA-G	NM_001102416	KNG
		NM_001801	CDO1	NM_015204	THSD7A
		NM_002117	HLA-C	NM_003944	SELENBP1
		NM_002123	HLA-DQB1	NM_030940	MGC4276
		NM_006120	HLA-DMA	NM_001013439	FXR1
		NM_001008397	ARHGEF1	NM_001171814	OAT
		NM_016582	PHT2		
		NM_001759	CCND2		
		NM_013279	C11orf9		
		NM_001425	EMP3		
		NM_001270987	LTBR		
		NM_000014	A2M		
22	**Liver**	NM_000014	A2M	NM_000346	SOX9
		NM_000055	BCHE	NM_000674	ADORA1
		NM_000096	CP	NM_000732	CD3D
		NM_000106	CYP2D6	NM_000876	IGF2R
		NM_000325	PITX2	NM_000903	NQO1
		NM_000412	HRG	NM_001168	BIRC5
		NM_000488	SERPINC1	NM_001255	CDC20
		NM_000583	GC	NM_001274	CHEK1
		NM_000669	ADH1C	NM_001305	CLDN4
		NM_000670	ADH4	NM_001311	CRIP1
		NM_000773	CYP2E1	NM_001767	CD2
		NM_001151	SLC25A4	NM_001803	CD52
		NM_001266	CES1	NM_001928	CFD
		NM_001385	DPYS	NM_002068	GNA15
		NM_001406	EFNB3	NM_002121	HLA-DPB1
		NM_001542	IGSF3	NM_002122	HLA-DQA1
		NM_001966	EHHADH	NM_002122	HLA-DQA2
		NM_001995	ACSL1	NM_002252	KCNS3
		NM_002080	GOT2	NM_002339	LSP1
		NM_002335	LRP5	NM_002423	MMP7
		NM_002591	PCK1	NM_002462	MX1
		NM_002998	SDC2	NM_002658	PLAU
		NM_003049	SLC10A1	NM_002961	S100A4
		NM_003251	THRSP	NM_002985	CCL5
		NM_003273	TM7SF2	NM_003004	SECTM1
		NM_003381	VIP	NM_003282	TNNI2
		NM_003745	SOCS1	NM_003975	SH2D2A
		NM_003986	BBOX1	NM_004079	CTSS
		NM_004068	AP2M1	NM_004131	GZMB
		NM_004171	SLC1A2	NM_004217	AURKB
		NM_004476	FOLH1	NM_004931	CD8B
		NM_005459	GUCA1C	NM_005601	NKG7
		NM_005622	ACSM3	NM_005602	CLDN11
		NM_005802	TOPORS	NM_005608	PTPRCAP
		NM_005989	AKR1D1	NM_005739	RASGRP1
		NM_006006	ZBTB16	NM_005849	IGSF6
		NM_006259	PRKG2	NM_005874	LILRB2
		NM_006514	SCN10A	NM_006144	GZMA
		NM_006684	CFHR4	NM_006573	TNFSF13B
		NM_006741	PPP1R1A	NM_006607	PTTG2
		NM_007021	C10orf10	NM_007057	ZWINT
		NM_007065	CDC37	NM_012189	CABYR

		NM_012190	ALDH1L1	NM_013269	CLEC2D
		NM_014160	MKRN2	NM_013308	GPR171
		NM_014343	CLDN15	NM_014442	SIGLEC8
		NM_014634	PPM1F	NM_016079	CHMP3
		NM_014885	ANAPC10	NM_016577	RAB6B
		NM_015460	MYRIP	NM_017850	C1orf109
		NM_016527	HAO2	NM_017899	TESC
		NM_017460	CYP3A4	NM_017915	PARPBP
		NM_017545	HAO1	NM_018098	ECT2
		NM_017786	SYBU	NM_018136	ASPM
		NM_017888	ACSM5	NM_018265	INAVA
		NM_018412	ST7	NM_018284	GBP3
		NM_018706	DHTKD1	NM_021250	LILRA5
		NM_018960	GNMT	NM_022089	ATP13A2
		NM_020061	OPN1LW	NM_024306	FA2H
		NM_020665	TMEM27	NM_024745	SHCBP1
		NM_021023	CFHR3	NM_031311	CPVL
		NM_021100	NFS1	NM_031471	FERMT3
		NM_021116	ADCY1	NM_032048	EMILIN2
		NM_021647	MFAP3L	NM_032117	MND1
		NM_024096	DCTPP1	NM_033119	NKD1
		NM_024861	C2orf54	NM_080668	CDCA5
		NM_031157	HNRNPA1	NM_130807	MOB3A
		NM_032601	MCEE	NM_139018	CD300LF
		NM_033507	GCK	NM_144584	HENMT1
		NM_052968	APOA5	NM_147130	NCR3
		NM_139055	ADAMTS15	NM_148170	CTSC
		NM_139160	DEPDC7	NM_152369	SLC44A3
		NM_144650	ADHFE1	NM_152573	RASEF
		NM_144962	PEBP4	NM_153206	JAML
		NM_152496	MANEAL	NM_170699	GPBAR1
		NM_152914	NATD1	NM_173574	ZNF683
		NM_172375	KCNH5	NM_198517	TBC1D10C
		NM_206827	RASL11A	NM_198952	NUDT1
				NM_207519	ZAP70
23	**Ischemic Heart (L. Ventricle)**	NM_000270	PNP	NM_000217	KCNA1
		NM_000345	SNCA	NM_001004464	OR10G8
		NM_000347	SPTB	NM_001005176	SP140
		NM_000417	IL2RA	NM_001005326	OR4F6
		NM_000737	CGB3	NM_001114396	KLRD1
		NM_001003938	HBM	NM_001169118	STIM2
		NM_001008388	CISD2	NM_001171905	NUDT16
		NM_001077180	METTL9	NM_001185015	SP110
		NM_001078175	SLC29A1	NM_001198801	EIF4G3
		NM_001102402	PCTP	NM_001256420	MAPRE2
		NM_001127892	SALL1	NM_001256560	DNASE1L3
		NM_001136179	EGR2	NM_001256742	CNOT10
		NM_001146037	SLC14A1	NM_001271889	CIB2
		NM_001167601	NEU4	NM_001277201	SIGLEC7
		NM_001195396	ARL4A	NM_001281451	TRIM50
		NM_001199691	TMEM56-RWDD3	NM_001286076	RPA2
		NM_001206941	TCAF1	NM_001302746	FASLG
		NM_001242839	GUK1	NM_001317061	RIPK1
		NM_001242871	SLAIN1	NM_001859	SLC31A1
		NM_001286484	TMEM14B	NM_001962	EFNA5
		NM_001291780	RBM38	NM_002864	PZP

	NM_001293085	BPGM	NM_003171	SUPV3L1
	NM_001302678	ADORA3	NM_003650	CST7
	NM_001308187	GYPA	NM_004131	GZMB
	NM_001317783	TSTA3	NM_004271	LY86
	NM_001317814	SLC25A37	NM_004398	DDX10
	NM_001318062	DBF4	NM_004568	SERPINB6
	NM_001318330	MINDY3	NM_004698	PRPF3
	NM_001320042	ISCU	NM_005103	FEZ1
	NM_001320321	TMTC2	NM_005210	CRYGB
	NM_001322239	BCL2L1	NM_005517	HMGN2
	NM_001322966	OAT	NM_006320	PGRMC2
	NM_001330173	CDKN3	NM_014280	DNAJC8
	NM_001442	FABP4	NM_014381	MLH3
	NM_001485	GBX2	NM_014471	SPINK4
	NM_001828	CLC	NM_014824	FCHSD2
	NM_002638	PI3	NM_015138	RTF1
	NM_002824	PTMS	NM_015621	CCDC69
	NM_003064	SLPI	NM_016024	RBMX2
	NM_003126	SPTA1	NM_016584	IL23A
	NM_003558	PIP5K1B	NM_016594	FKBP11
	NM_003696	OR6A2	NM_020395	INTS12
	NM_003851	CREG1	NM_024312	GNPTAB
	NM_004359	CDC34	NM_032292	GON4L
	NM_005326	HAGH	NM_032522	ZBTB37
	NM_007111	TFDP1	NM_033135	PDGFD
	NM_007285	GABARAPL2	NM_080390	TCEAL2
	NM_012089	ABCB10	NM_130900	RAET1L
	NM_012179	FBXO7	NM_138287	DTX3L
	NM_013339	ALG6	NM_144573	NEXN
	NM_014331	SLC7A11	NM_152626	ZNF92
	NM_014399	TSPAN13	NM_152649	MLKL
	NM_015999	ADIPOR1	NM_153263	ZNF549
	NM_016016	SLC25A39	NM_177423	PPFIA1
	NM_016068	FIS1	NM_178831	GATS
	NM_016353	ZDHHC2	NM_181351	NCAM1
	NM_016633	AHSP	NM_182709	KAT5
	NM_018270	MRGBP	NM_198562	C3orf62
	NM_018457	PRR13	NM_207312	TUBA3E
	NM_020244	CHPT1	NM_207422	NHSL2
	NM_030650	LNPK	NM_001010871	ADCY10P1
	NM_032344	NUDT22	NM_003934	GTF2IRD1P1
	NM_052831	SLC18B1	NM_026791	HCG27
	NM_052874	STX1B	NM_032768	STK16
	NM_144563	RPIA	NM_006961	ZNF19
	NM_144665	SESN3	NM_024610	HSPBAP1
	NM_145238	ZSCAN20	NM_018326	GIMAP4
	NM_144624	UHMK1	NM_152379	C1orf131
	NM_032291	SGIP1	NM_000449	RFX5
	NM_144660	SAMD8	NM_003449	TRIM26
	NM_153811	SLC38A6	NM_005612	REST
	NM_003304	TRPC1	NM_020811	CARNS1
	NM_003805	CRADD	NM_016466	ANKRD39
	NM_198149	SHISA4	NM_000333	ATXN7
	NM_194291	TMEM65	NM_033355	CASP8

24	**Parkinson's (M)**	NM_005058	RBM	NM_007262	PARK7
		NM_001670	ARVCF	NM_001069	TUBB2A
		NM_000498	CYP11B2	NM_004408	DNM1
		NM_173728	ARHGEF15	NM_207521	RTN4
		NM_004381	CREBL1	NM_000988	RPL27
		NM_000061	BTK	NM_001024	RPS21
		NM_004183	BEST1	NM_001280	CIRBP
		NM_016151	TAOK2	NM_005004	NDUFB8
		NM_014792	KIAA0125	NM_001677	ATP1B1
		NM_001645	APOC1	NM_198829	RAC1
		NM_003170	SUPT6H	NM_001402	EEF1A1
		NM_031288	ZNHIT4	NM_003973	RPL14
		NM_001240	CCNT1	NM_003795	SNX3
		NM_024840	ZNF613	NM_012286	MORF4L2
		NM_024791	PDZD3	NM_002796	PSMB4
		NM_031305	ARHGAP24	NM_000969	RPL5
		NM_016929	CLIC5	NM_016091	EIF3S6IP
		NM_139049	MAPK8	NM_178012	TUBB2B
		NM_002688	GP1BB	NM_006123	IDS
		NM_003394	WNT10B	NM_004052	BNIP3
		NM_001918	DBT	NM_004546	NDUFB2
		NM_006771	KRT38	NM_206917	NGFRAP1
		NM_181869	APAF1	NM_001690	ATP6V1A
		NM_018156	VPS13D	NM_002300	LDHB
		NM_018664	SNFT	NM_000986	RPL24
		NM_017675	PCLKC	NM_153201	HSPA8
		NM_032459	EFS	NM_001867	COX7C
		NM_022778	CCDC21	NM_000812	GABRB1
		NM_014773	KIAA0141	NM_006317	BASP1
		NM_002319	LRCH4	NM_001011	RPS7
		NM_001986	ETV4	NM_031157	HNRPA1
		NM_080603	ZSWIM1	NM_001614	ACTG1
		NM_183395	NLRP3	NM_000454	SOD1
		NM_052819	CARD14	NM_005348	HSP90AA1
		NM_001695	ATP6V1C1	NM_004545	NDUFB1
		NM_005629	DUX4	NM_001696	ATP6V1E1
		NM_001017535	VDR	NM_000989	RPL30
		NM_172390	NFATC1	NM_199440	HSPD1
		NM_172390	NFATC1	NM_015710	GLTSCR2
		NM_020638	FGF23	NM_005102	FEZ2
		NM_014626	TAAR2	NM_031210	C14orf156
		NM_020407	RHBG	NM_000291	PGK1
		NM_182611	GPR144	NM_004859	CLTC
		NM_004444	EPHB4	NM_002567	PEBP1
		NM_005734	HIPK3	NM_015085	GARNL4
		NM_018836	AJAP1	NM_000944	PPP3CA
		NM_021220	OVOL2	NM_213725	RPLP1
		NM_024787	RNF122	NM_001689	ATP5G3
		NM_013938	OR10H3	NM_006936	SUMO3
		NM_021046	KRTAP5-8	NM_020686	ABAT
		NM_175085	GART	NM_001967	EIF4A2
		NM_005406	ROCK1	NM_001997	FAU
		NM_002885	RAP1GAP	NM_001099693	RPL31
		NM_003580	NSMAF	NM_020531	C20orf3
		NM_007346	OGFR	NM_002032	FTH1
		NM_013992	PAX8	NM_004046	ATP5A1

		NM_172164	NASP	NM_001032	RPS29
		NM_001771	CD22	NM_016127	TMEM66
		NM_201597	CACNB2	NM_003746	DYNLL1
		NM_000890	KCNJ5	NM_001023	RPS20
		NM_005225	E2F1	NM_005235	ERBB4
		NM_022755	IPPK	NM_006283	TACC1
		NM_198830	ACLY	NM_001386	DPYSL2
		NM_024949	WWC2	NM_207578	KIAA1245
		NM_025256	EHMT2	NM_207578	PRKACB
		NM_017621	ALKBH4	NM_004549	NDUFC2
		NM_023003	TM6SF1	NM_000997	RPL37
		NM_003874	CD84	NM_014394	GHITM
		NM_199204	DHRS9	NM_003295	TPT1
		NM_003864	SAP30	NM_203431	PPIA
		NM_001045	SLC6A4	NM_006004	UQCRH
		NM_024756	MMRN2	NM_001009993	LOC130074
		NM_181710	ZNRF4	NM_001861	COX4I1
		NM_021724	NR1D1	NM_006464	TGOLN2
		NM_030776	ZBP1	NM_001035258	RPL38
25	**Parkinson's (F)**	NM_033375	MYO1C	NM_033481	FBXO9
		NM_203458	NOTCH2NL	NM_005561	LAMP1
		NM_001338	CXADR	NM_001659	ARF3
		NM_001918	DBT	NM_002567	PEBP1
		NM_020791	TAOK1	NM_005503	APBA2
		NM_003906	MCM3AP	NM_024293	FAM134A
		NM_017590	ZC3H7B	NM_000374	UROD
		NM_002547	OPHN1	NM_003928	FAM127A
		NM_021599	ADAMTS2	NM_018640	LMO3
		NM_005817	M6PRBP1	NM_022648	TNS1
		NM_014736	KIAA0101	NM_174929	ZMIZ2
		NM_001039145	WBSCR23	NM_014850	SRGAP3
		NM_017460	CYP3A4	NM_004894	C14orf2
		NM_016426	GTSE1	NM_014747	RIMS3
		NM_005697	SCAMP2	NM_004859	CLTC
		NM_017658	KLHL28	NM_016841	MAPT
		NM_020117	LARS	NM_001696	ATP6V1E1
		NM_001670	ARVCF	NM_012137	DDAH1
		NM_003123	SPN	NM_206857	RTN1
		NM_018404	CENTA2	NM_014206	C11orf10
		NM_018845	RAG1AP1	NM_005389	PCMT1
		NM_019041	MTRF1L	NM_058199	OLFM1
		NM_009586	SIM2	NM_024165	PHF1
		NM_006883	SHOX	NM_031298	TMEM93
		NM_003048	SLC9A2	NM_001024	RPS21
		NM_003048	SLC9A2	NM_002074	GNB1
		NM_017908	ZNF446	NM_001040455	SIDT2
		NM_001645	APOC1	NM_020310	MNT
		NM_078630	MSL3L1	NM_007326	CYB5R3
		NM_014139	SCN11A	NM_145752	CDIPT
		NM_024804	ZNF669	NM_005137	DGCR2
		NM_003172	SURF1	NM_020481	ANK1
		NM_138292	ATM	NM_014452	TNFRSF21
		NM_012098	ANGPTL2	NM_022910	NDRG4
		NM_006134	TMEM50B	NM_012319	SLC39A6
		NM_001559	IL12RB2	NM_001035258	RPL38
		NM_003294	TPSAB1	NM_001012452	GOLGA8E

	NM_012197	RABGAP1	NM_014754	PTDSS1
	NM_002173	IFNA16	NM_025222	TMEM113
	NM_002348	LY9	NM_001039703	NBPF10
	NM_018994	FBXO42	NM_201559	FOXO3
	NM_139045	SMARCA2	NM_006004	UQCRH
	NM_001048199	RCC1	NM_005235	ERBB4
	NM_021652	SMA3	NM_016127	TMEM66
	NM_002105	H2AFX	NM_001478	B4GALNT1
	NM_007344	TTF1	NM_015294	TRIM37
	NM_004332	BPHL	NM_012202	GNG3
	NM_207519	ZAP70	NM_006695	RPIP8
	NM_000168	GLI3	NM_002045	GAP43
	NM_014381	MLH3	NM_013365	GGA1
	NM_001531	MR1	NM_006122	MAN2A2
	NM_020407	RHBG	NM_014954	RPH3A
	NM_014371	AKAP8L	NM_033546	MRLC2
	NM_004707	ATG12	NM_006598	SLC12A7
	NM_005378	MYCN	NM_183243	IMPDH1
	NM_170720	KCNJ14	NM_006646	WASF3
	NM_012433	SF3B1	NM_178124	CXorf40A
	NM_177963	SYT12	NM_152374	FLJ38984
	NM_019895	CLDND1	NM_175932	PSMD13
	NM_025232	REEP4	NM_007184	NISCH
	NM_001626	AKT2	NM_016424	CROP
	NM_016261	TUBD1	NM_006839	IMMT
	NM_006108	SPON1	NM_002073	GNAZ
	NM_001417	EIF4B	NM_170663	MINK1
	NM_024733	ZNF665	NM_181738	PRDX2
	NM_199439	PRDM10	NM_138736	GNAO1
	NM_182686	KIAA0319L	NM_006701	TXNL4A
	NM_014508	APOBEC3C	NM_002744	PRKCZ
	NM_021192	HOXD11	NM_007158	CSDE1
	NM_003199	TCF4	NM_004809	STOML1
	NM_005490	SH2D3A	NM_032920	PDXK
	NM_000748	CHRNB2	NM_001627	ALCAM
	NM_001494	GDI2	NM_014399	TSPAN13
	NM_002127	HLA-G	NM_015458	MTMR9
	NM_021184	C6orf47	NM_182661	CERK
